# Limitations of the Goligher classification in randomized trials for hemorrhoidal disease: a qualitative systematic review of selection criteria

**DOI:** 10.1007/s10151-025-03170-y

**Published:** 2025-06-10

**Authors:** J. Y. van Oostendorp, U. Grossi, I. Hoxhaj, M. L. Kimman, S. Z. Kuiper, S. O. Breukink, I. J. M. Han-Geurts, G. Gallo

**Affiliations:** 1 Department of Surgery, Amsterdam UMC, Location AMC, Amsterdam, The Netherlands; 2 Department of Surgery, Proctos Kliniek, Bilthoven, The Netherlands; 3 Department of Surgery, Oncology and Gastroenterology-DiSCOG, University of Padova, Padua, Italy; 4 Department of Clinical Epidemiology and Medical Technology Assessment, Care and Public Health, Research Institute (CAPHRI), Maastricht University Medical Centre, Maastricht, The Netherlands; 5 Department of Surgery, School of Nutrition and Translational Research in Metabolism (NUTRIM), Maastricht University, Maastricht, The Netherlands; 6 Department of Surgery, School for Oncology and Reproduction (GROW), Maastricht University, Maastricht, The Netherlands; 7 Department of Surgery, Sapienza University of Rome, Rome, Italy; 8 Surgery Unit 2, Regional Hospital Treviso, Piazzale dell’Ospedale 1, 31100 Treviso, Italy

**Keywords:** Hemorrhoids, Hemorrhoidal disease, Goligher classification, Patient selection, Randomized controlled trials, Classification systems

## Abstract

**Background:**

The diverse range of therapeutic options for hemorrhoidal disease (HD) highlights the need for precise classification systems to guide treatment. Although the Goligher classification remains the most widely used, it has been criticized for its simplicity and limited ability to capture symptom severity or guide treatment decisions. This study aims to evaluate the patient selection criteria and classification systems employed in randomized controlled trials (RCTs) for HD.

**Methods:**

A systematic review was conducted following the 2020 PRISMA guidelines. A comprehensive search of databases identified randomized controlled trials (RCTs) comparing treatments for HD, focusing on classification systems used for patient enrollment. Eligible studies included adult patients and at least one arm involving surgical treatment.

**Results:**

Out of 6692 records, 162 studies met the inclusion criteria, with a median cohort size of 84 patients and 55.4% male. Most studies (86.4%) used the Goligher system, though the majority did not fully describe or cite the system. Only 13.6% of studies employed more recent alternative classification systems. The most common outcome measures across studies were postoperative pain (147 studies) and complications (133 studies). Recurrence rates were reported in 42% of studies, yet 70% of these did not provide adequate inclusion criteria or references to Goligher’s classification.

**Conclusions:**

The inconsistent application of the Goligher classification and the variability in patient selection criteria across RCTs highlight the need for more nuanced and standardized systems. Future research should focus on refining classification methods and incorporating patient-reported outcomes to improve the reliability and relevance of HD trials.

**PROSPERO registration:**

CRD42023387339.

**Supplementary Information:**

The online version contains supplementary material available at 10.1007/s10151-025-03170-y.

## Introduction

Hemorrhoidal disease (HD) poses a substantial clinical burden, affecting up to 39% of the general population [1, 2]. The most commonly used grading system proposed by Goligher in the 1970s is primarily based on the degree of prolapse [3]. While this system is widely adopted in both clinical practice and research, it overlooks several key aspects, such as the number of affected piles and critical symptoms like anal bleeding, itching, pain, and soiling, as well as their impact on quality of life [4].

The limitations of the Goligher system create challenges in evaluating and comparing treatment approaches. Patients’ subjective experiences often do not align with the grades of prolapse described by Goligher, making it difficult to accurately assess disease severity [5]. Moreover, the classification is limited to prolapsing internal hemorrhoids, disregarding mixed hemorrhoids (which involve both internal and external components) and other specific scenarios, such as circumferential prolapse or thrombosis [4, 6]. These shortcomings have been highlighted in practice guidelines, such as those from the American Society of Colon and Rectal Surgeons (ASCRS), which emphasize the importance of a comprehensive evaluation beyond prolapse grading [7]. The ASCRS guidelines advocate for a detailed assessment of symptom severity, bleeding patterns, and associated conditions to inform treatment decisions. Therefore, there is an increasing need for a more inclusive and nuanced classification system that can capture the full range of clinical presentations in HD [8].

Previous studies, including those conducted by our research group, have shown that healthcare providers demonstrate poor inter-rater reliability when using the Goligher system, further raising concerns about its clinical and research utility [9]. This inconsistency poses challenges, particularly in research settings where precise classification is essential for patient inclusion and outcome evaluation [10]. The gap between prolapse severity and symptom burden also limits the system’s ability to guide effective treatment decisions and has led researchers to seldom use changes in Goligher’s grade as primary endpoints in clinical trials [9]. As a result, researchers have often opted to use more patient-centered outcomes, such as patient-reported outcomes or clearly defined endpoints like recurrence, rather than relying solely on changes in Goligher’s grade.

An area of particular interest in HD research is the selection criteria employed in randomized clinical trials (RCTs). These criteria are crucial as they directly impact the validity of trial outcomes and, subsequently, the evidence base for treatment efficacy. However, it appears that the classification systems used to enroll participants in these trials may vary, complicating the comparison of results.

The aim of this qualitative systematic review is to provide a comprehensive summary of the selection criteria and classification systems used for the enrollment of patients in RCTs comparing treatments for HD. By synthesizing the existing literature, we aim to identify patterns, trends, and areas of variation in the criteria used across different studies. Ultimately, this review seeks not only to enhance understanding of the current landscape of HD classification but also to serve as a foundation for the development of a more inclusive and clinically relevant classification system in the future.

## Methods

The protocol for this systematic review was developed with pre-specified methods of analysis and eligibility criteria in accordance with the 2020 Preferred Reporting Items for Systematic Reviews and Meta-Analyses (PRISMA) guidance [11]. The protocol was prospectively registered with PROSPERO (CRD42023387339).

### Study characteristics

Search term definitions were deliberately inclusive to facilitate a comprehensive search of studies reporting HD in adults aged over 18 years. Eligible studies were RCTs that evaluated treatments for HD with at least one study arm involving a surgical procedure. Our decision to exclude RCTs involving solely office-based interventions like sclerotherapy or rubber band ligation was aimed at ensuring the inclusion of patients with more severe HD. This is particularly relevant because evidence suggests that inter-rater agreement on Goligher classification tends to be poorer for grades III [9], making it crucial to focus on studies involving more severe cases to better assess the classification system’s reliability and utility in a clinical setting. Eligible studies included those examining outpatient procedures such as rubber band ligation, sclerotherapy, infrared photo-coagulation, cryotherapy, as well as surgical procedures like open or closed hemorrhoidectomy, hemorrhoidopexy, stapled hemorrhoidectomy, hemorrhoidal artery ligation and recto-anal repair, and transanal hemorrhoidal dearterialization. Full-text articles needed to be available in the English language.

Studies were excluded if they were non-randomized, observational, case series, case reports, IDEAL 1 and 2a studies focusing on proof of concept or safety of new surgical materials or techniques, study protocols, superseded series, or studies focused solely on treatment of postoperative outcomes (i.e., pain) or anesthesia methods [12]. Additionally, studies including patients with concomitant proctologic abnormalities (e.g., fistulas, perianal Crohn’s disease, fissures) were excluded. Only peer-reviewed publications reporting primary data were considered, while reviews, meta-analyses, editorials, letters, conference abstracts, and proceedings were excluded during screening.

### Information sources and study selection

A comprehensive literature search was conducted using PubMed, MEDLINE, EMBASE, Web of Science, and the Cochrane Library. Common search terms related to HD classification were employed (Appendix 1). Reference lists of the included studies were also hand-searched to identify additional relevant studies. Studies published up to final search date of October 19, 2023 were eligible for inclusion.

### Data extraction

Two reviewers independently and systematically reviewed the results, performing a title screen followed by an abstract screen. Any differences in the reviewers’ assessments were resolved by consensus. Subsequently, two reviewers independently and in duplicate abstracted data on studies and selection criteria relevant to patient, physician, and context characteristics. Data extraction included details on the first author, publication year, country of origin, study design, single or multicenter nature of the study, study duration (months), total number of patients, number and proportion of male patients, types of procedures (surgical or office-based), number of study arms, treatments in each study arm, number of patients per treatment arm, type of treatment in the control arm, number of patients in the control arm, follow-up duration (months), main study outcomes, authors’ conclusions of these study outcomes, type of classification system for HD, and use of criteria or scoring systems for HD, including their range and whether they covered structural or functional characteristics.

Assessing the risk of bias was not applicable for this systematic review, as its purpose was to provide an overview of the classification systems or selection criteria used to select patients eligible for clinical trials, without a quality assessment of quantitative outcome data. Data were extracted into a structured Microsoft Excel spreadsheet (XP professional edition; Microsoft Corp, Redmond, Washington, USA).

### Strategy for data synthesis

A narrative synthesis of the findings from selected studies was conducted, focusing primarily on the classification systems and criteria used for patient selection in clinical trials, rather than the trial outcomes themselves. Our analysis emphasized patient, physician, and contextual characteristics influencing the inclusion of patients in studies, specifically examining how these factors impacted the use of the Goligher system or other classification criteria. Although many studies reported a wide range of outcomes—such as recurrence, postoperative pain, complications (including bleeding, incontinence, stenosis, etc.), symptom control, and patient satisfaction—our primary interest was not in these clinical outcomes but in how patients were classified for trial inclusion. Since most studies did not clearly distinguish between primary and secondary endpoints, we summarized the reported outcomes descriptively, while maintaining our main focus on the classification and criteria data relevant to our research question.

## Results

### Study selection

**Fig. 1:**
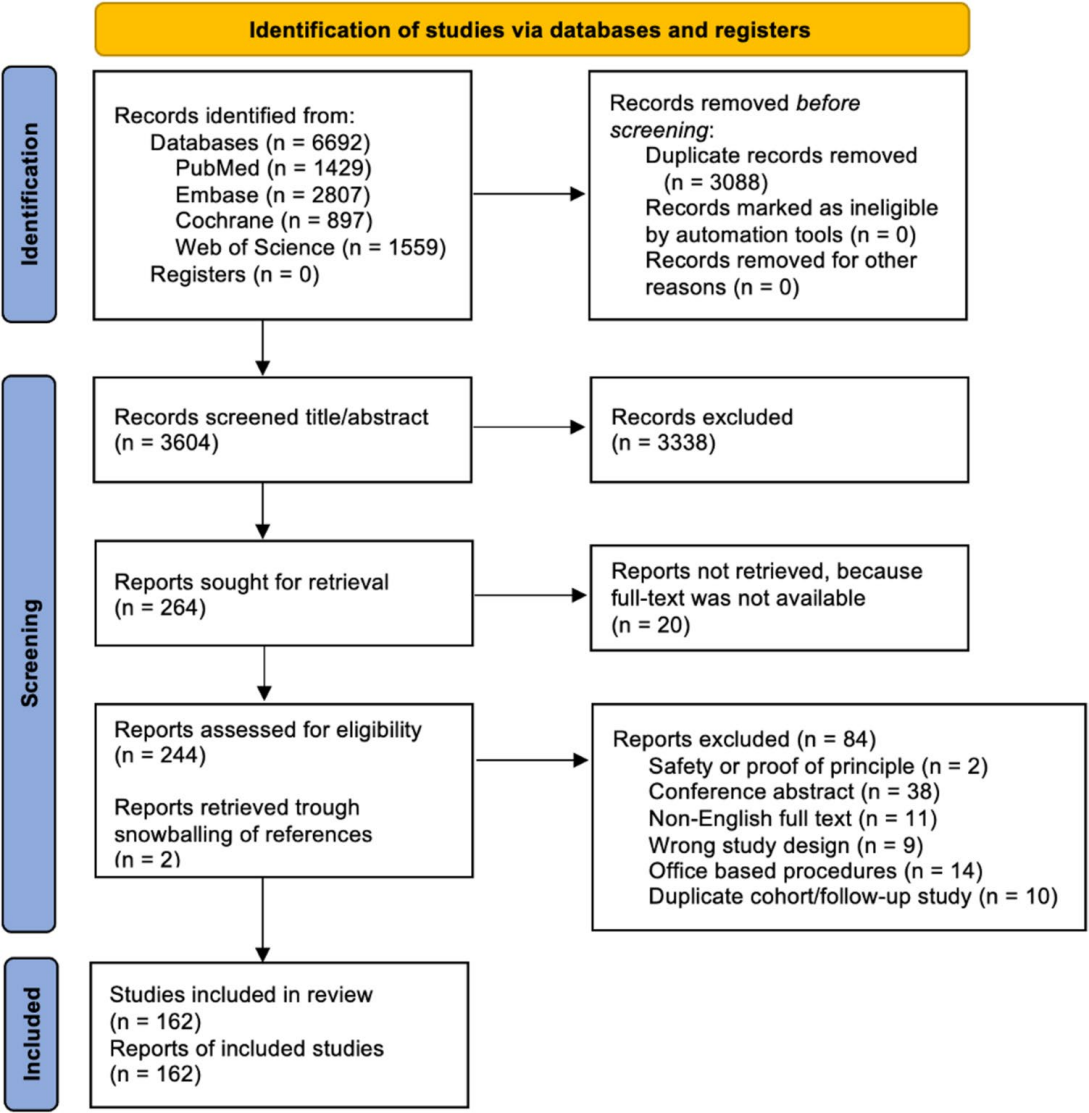
PRISMA diagram

Out of a total of 6692 records, 3088 duplicates were removed. The remaining 3604 records were screened by title and abstract, resulting in the exclusion of 3338 records (92.6%). The full text of 20 reports could not be retrieved. The remaining 244 reports were assessed for eligibility, and 84 (34.4%) were excluded for not meeting the inclusion criteria, with the reasons listed in Fig. 1. An additional two reports meeting the inclusion criteria were identified through snowballing references from included studies. In total, 162 studies published between 1980 and 2023 met the inclusion criteria and were analyzed in this review.

### Demographic and clinical characteristics

**Fig. 2:**
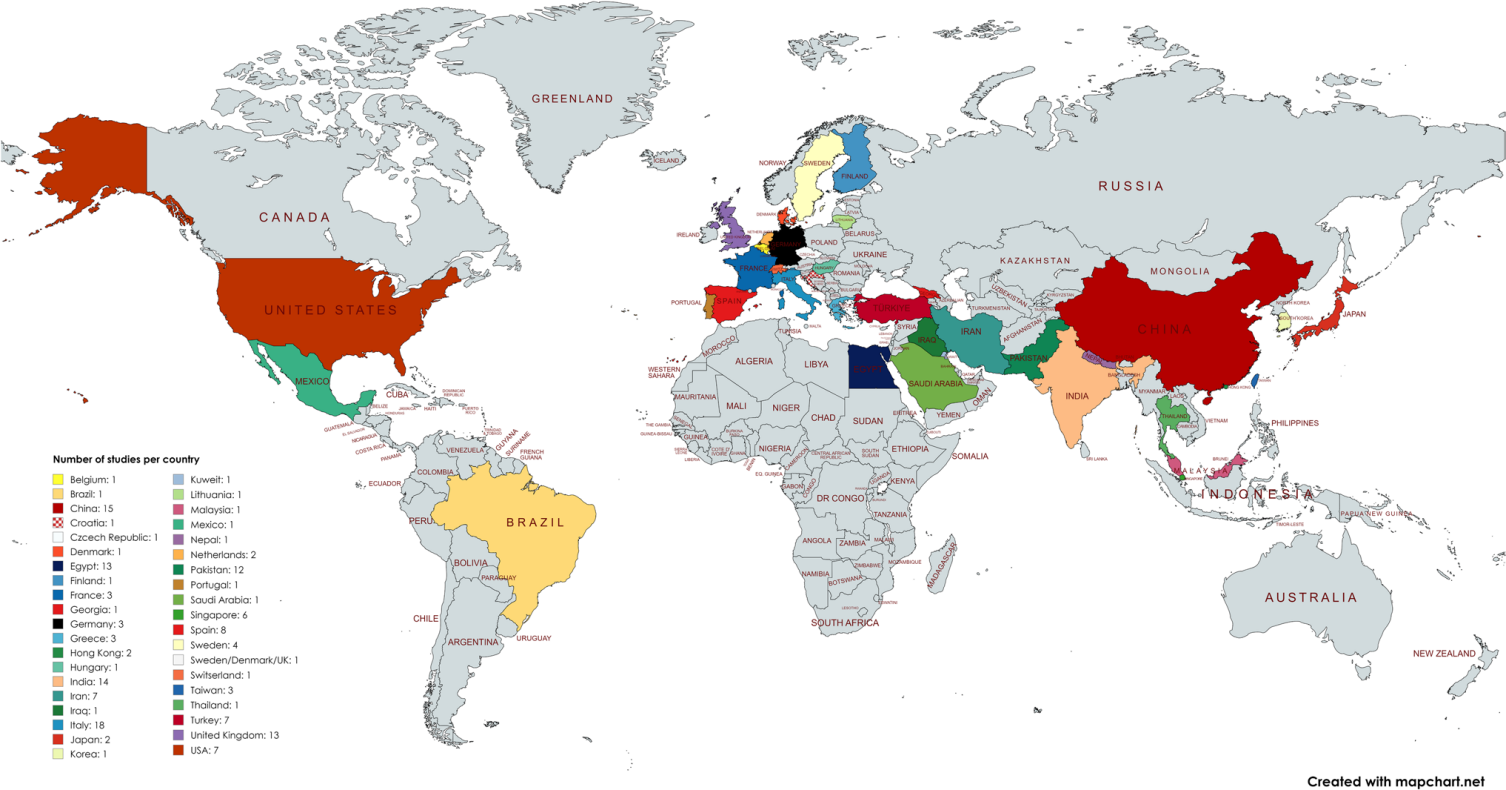
Map chart indicating the geographic distribution of the studies

The 162 studies originated from 38 different countries (Table 1; Fig. 2). The six most represented countries were Italy (*n* = 18), China (*n* = 15), India (*n* = 14), the UK (*n* = 13), Egypt (*n* = 13), and Pakistan (*n* = 12). Nineteen (11.7%) studies were multicenter. The median recruitment duration was 20 months (IQR 12–28). The median cohort size was 84 patients (IQR 50–130). Gender distribution was reported in 153 (94.4%) of the studies. Of the total 18,667 patients, 10,346 (55.4%) were male. Most studies (95.1%) compared two different treatments, while eight studies (4.9%) compared three different treatments. The median follow-up time in the studies was 12 months (IQR 3–12), with a range from 1 day to 9.5 years.

**Table 1:** Overview of randomized controlled trials included in this review

	Total studies (*N* = 162)
Geographic distribution,* N* (%)	
Asia	69 (43)
Europe	50 (31)
Middle east	21 (13)
Africa	13 (8)
North America	8 (5)
South America	1 (1)
Single center, *N* (%)	143 (88)
Multicenter, *N* (%)	19 (12)
Time horizon (months)	
Mean (SD)	22.3 (17.4)
Median (min–max), IQR	20 (2–155), IQR 12–28
Cohort size (no. patients)	
Mean (SD)	115.2 (109.9)
Median (min–max), IQR	84 (20–777), IQR 50–130
Number of treatment arms, *N* (%)	
2 treatment arms	154 (95.1)
3 treatment arms	8 (4.9)
Follow-up time (months)	
Mean (SD)	13.1 (16)
Median (min–max), IQR	12 (1 day–114 months), IQR 3–12
Comparisons within studies, *N* (%)	
Excisional vs. excisional	78 (48)
Excisional vs. non-excisional	45 (28)
Non-excisional vs. non-excisional	27 (17)
Office-based vs. excisional	7 (4)
Office-based vs. non-excisional	6 (4)
Classification criteria, *N* (%)	
A. Classification systems	
Goligher	140 (86)
Nivatvongs	2 (1)
Gerjy/Nyström	2 (1)
Milles	1 (1)
Banov	1 (1)
B. Other criteria	
Structural	16 (10)
Symptomatic	6 (3.7)
Surgical candidacy	3 (1.9)
Failed previous treatment	4 (2.5)
Structural	2 (1.2)
Not reported	1 (0.6)

### Use of Goligher’s classification system

The vast majority (86.4%) of the studies used the Goligher classification system as criteria for patient selection (Table 1). However, 103 of these studies (73.6%) only mentioned grades II, III, or IV, or mixed hemorrhoids, without specifically naming Goligher, adequately defining the grading, or referencing Goligher’s published work [3]. Of the 140 reports using the Goligher classification, only 19 (13.6%) adequately referenced and mentioned the Goligher in their methods section where the inclusion criteria for patient selection were described. Additionally, six reports (4.3%) mentioned Goligher in the methods section without referencing the published work. In another six reports (4.3%), Goligher was mentioned in the introduction of the report, so it could be assumed that the grading in the methods or results section followed the Goligher classification system. In two reports, Goligher was adequately mentioned and referenced, but only in the results section. Four reports (2.9%) did not mention Goligher at all but fully described the grades and their definitions in the methods section, clearly indicating the patients selected for enrollment.

### Use of other classification systems

In the 22 RCTs (13.6%) that did not reference the Goligher classification or the “grade” of hemorrhoids, various other inclusion criteria or classification systems were used for patient selection (Table 1). For example, two Italian studies utilized the Nivatvongs classification system, which not only assesses the structural prolapse similar to Goligher’s but also incorporates bleeding symptoms [13, 14]. In their RCT comparing diathermy hemorrhoidectomy with stapled anopexy, Gerjy et al. [15] proposed a new classification system, combining three items: (1) patient self-report of prolapse requiring manual reposition; (2) surgeon assessment of prolapse when the patient negated manual reposition; and (3) surgeon evaluation of the external component. This system categorizes prolapse as 1–3 (1 = no prolapse; 2 = spontaneously reducing prolapse; 3 = prolapse requiring manual repositioning) and the external component as A–C (A = no external component; B = one or few tags, C = circumferential). Subsequently, a large French study also adopted this classification system for patient inclusion [16]. Additionally, a 2002 study from Switzerland employed the Milles classification system [17], while a Chinese study selected patients on the basis of a clinical diagnosis of mixed hemorrhoids (veins above and below dental line) and Banov’s classification of grades III–IV internal hemorrhoids or symptomatic external hemorrhoids [18].

### Studies without clear classification methodology

In 16 studies that did not explicitly reference the Goligher system or any other predefined classification systems, and did not use the term “grade” in their inclusion criteria, the criteria were based on a range of symptomatic and anatomical characteristics, along with patients’ treatment histories [19–34] (Table 1).

First, several studies focused on symptomatic and prolapsing hemorrhoids, both with and without external hemorrhoids, presenting with symptoms such as bleeding, hygiene difficulties, or discomfort. Some studies specifically included patients with thrombosed hemorrhoids or large hemorrhoids prone to permanent prolapse. Irreducible prolapse was a common criterion, with studies including patients with “three irreducible prolapsing piles” or “symptomatic prolapsed irreducible hemorrhoids.” Circumferential mixed hemorrhoids were also considered as a selection criterion.

Second, surgical candidacy was a significant inclusion factor in some studies, i.e., patients requiring invasive surgical treatment for their hemorrhoids. Additionally, a subset of studies focused on patients who had failed previous treatments, such as rubber band ligation, were fit for anesthesia, and without treatment preference.

### Comparators across studies

Seventy-eight (48.1%) studies compared two or more excisional techniques (Table 1). Forty-five (27.8%) studies compared an excisional with a non-excisional surgical technique. Twenty-seven (16.7%) studies compared two non-excisional techniques, while seven (4.3%) compared an office-based procedure to an excisional surgical procedure. Six (3.7%) studies compared an office-based procedure to a non-excisional surgical procedure.

### Main RCT study outcomes

**Fig. 3:**
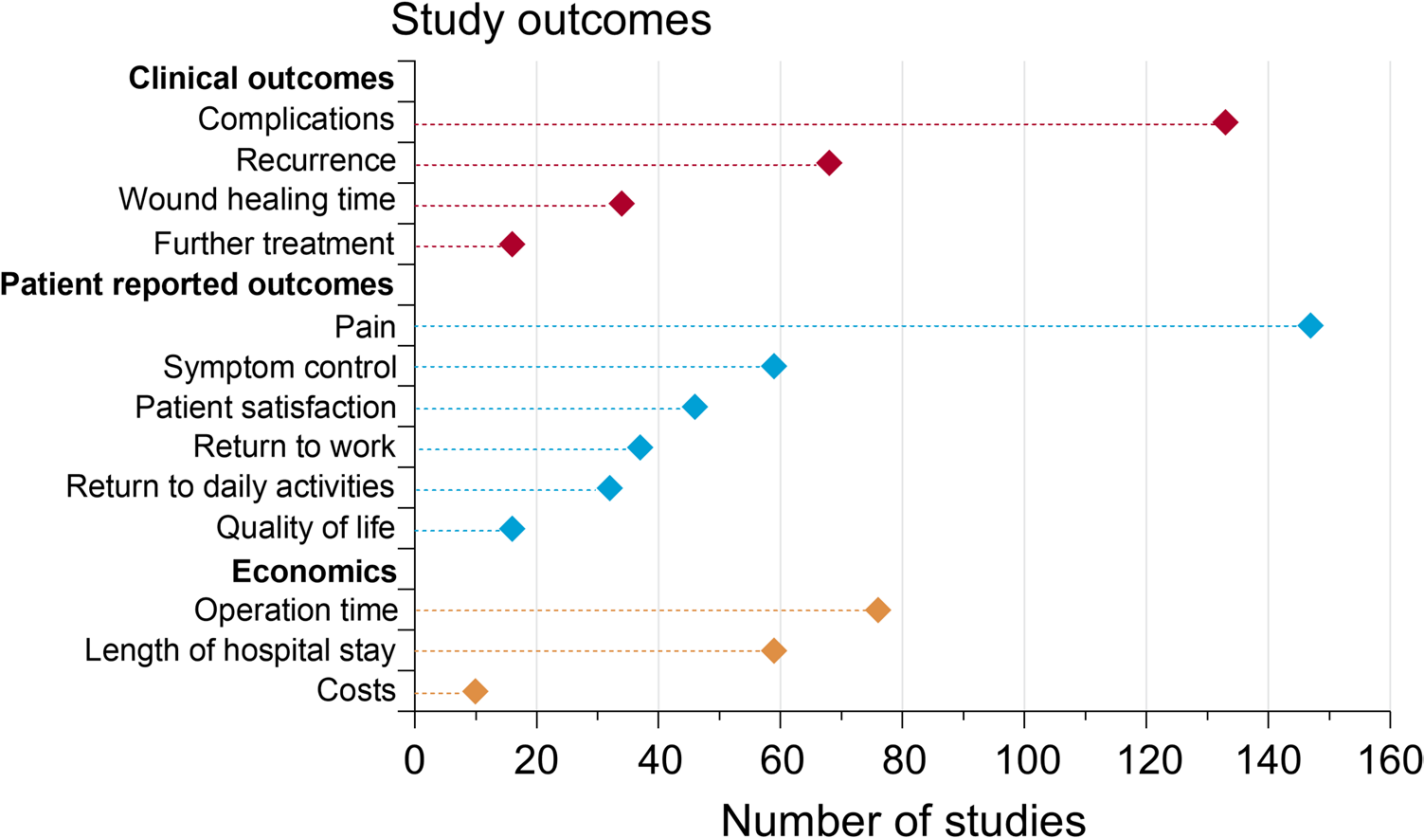
Graph summarizing the categories of the reported study outcomes

Across the 162 clinical trials, a wide range of outcomes were evaluated. These are summarized in Fig. 3, which indicates the number of studies reporting on each outcome category. The majority of studies focused on pain (147 studies) and complications (133 studies). Among the 68 (42%) studies reporting on recurrence rates, 48 either lack detailed inclusion criteria or fail to reference the Goligher classification. In contrast, the remaining 20 studies that include recurrence as an outcome either provide appropriate inclusion criteria or reference the Goligher classification.

## Discussion

Our qualitative systematic review aimed to provide a comprehensive overview of the selection criteria and classification systems used in RCTs comparing treatments for HD. We found that the majority of studies (86.4%) relied on the Goligher classification system for patient selection. However, only a small proportion of these studies adequately cited Goligher’s original definitions, with most merely listing the HD grades (I to IV) without providing precise definitions or appropriate references. Importantly, the Goligher system serves two distinct purposes that are often conflated: first, as a tool for selecting patients to participate in trials, and second, for evaluating outcomes such as recurrence. This dual role of the Goligher system underscores a need to emphasize clarity in its application across studies.

### Goligher classification: strengths and limitations

The Goligher classification is widely adopted owing to its simplicity and ability to categorize prolapsing hemorrhoids. However, it has several limitations, particularly its focus on prolapse without considering symptom burden. This issue has been widely recognized in the literature [5, 9, 35–37], as well as in the European Society of Coloproctology (ESCP) guideline for the management of hemorrhoidal disease [38]. The ESCP guideline emphasizes the importance of prioritizing symptom burden over anatomical classification in guiding treatment decisions and advocates for a more patient-centered approach that reflects the real-world impact of the disease.

Moreover, while the simplicity of the Goligher classification makes it advantageous in clinical communication, its lack of standardization across studies and poor interobserver agreement—especially for grades II and III—undermine its reliability [9]. This lack of standardization not only contributes to inconsistencies in patient selection but also increases the risk of heterogeneous study populations, complicating efforts to ensure uniformity across trials. These poorly defined selection criteria further exacerbate challenges in comparing study outcomes, particularly when the Goligher classification is inconsistently applied as both a patient selection tool and an outcome measurement system. Such inconsistencies underscore the need for clearer guidelines and more rigorous methodologies to improve the comparability and validity of research in this field.

### Goligher as outcome measurement

The Goligher system is also often used to assess recurrence and other post-treatment outcomes. However, the reliability of recurrence assessments is questionable if the initial classification of hemorrhoids is not consistently applied. Our previous survey [9] revealed poor interobserver agreement, further highlighting the need for more precise tools when classifying patients for both baseline and post-treatment evaluations. While the Goligher classification remains a practical tool for categorizing patients in clinical settings, its inconsistent—particularly when the grade assigned at the start of a study is unclear or inconsistently defined—undermines its reliability as an outcome measurement tool. For example, 48 out of 68 studies (70%) that aimed to assess recurrence failed to adequately describe their inclusion criteria or reference the Goligher classification when discussing HD grade. Given that reliable outcome assessment is crucial for understanding treatment efficacy, the inconsistent use of Goligher at both the selection and evaluation stages underscores the need for more rigorous methodologies and clearer reporting standards.

### Need for more comprehensive systems

To address the limitations of the Goligher classification, several authors have proposed alternative methods that integrate both anatomical findings and subjective symptoms to better reflect disease severity [10, 13, 17, 37]. Systems like the Single Pile Hemorrhoid Classification [5], which assesses each hemorrhoid individually, offer a more granular approach to patient assessment, enabling tailored treatment strategies for cases with varying degrees of severity. However, its complexity, compared to the simplicity of the Goligher classification, may limit its adoption in routine clinical practice.

Similarly, the recently proposed BPRST classification provides a novel, multidimensional framework by incorporating bleeding, prolapse, reduction, skin tags, and thrombosis [37]. By combining objective clinical findings with key symptomatic components of HD, the BPRST system enables a more nuanced characterization of disease severity and bridges the gap between anatomical classification and symptom burden. Nevertheless, its broader application in routine practice may face similar challenges, including complexity and the need for clinician familiarity.

These challenges underscore the need for systems that balance comprehensiveness with practicality. While moving away from intuitive systems like Goligher toward more complex alternatives presents practical difficulties [39], the integration of patient-reported outcomes (PROMs) offers a practical and patient-centered solution. PROMs, which capture the patient’s perspective on symptom burden and quality of life, could enhance existing classification systems by providing a more holistic understanding of the patient’s experience of the disease [35, 40–44].

### PROMs and outcome assessment

Several trials, such as the Hubble trial [42] and studies using the Hemorrhoidal Disease Symptom Score (HDSS) and Short Health Scale for Hemorrhoidal Disease (SHSHD) [41], have demonstrated the value of using PROMs to evaluate outcomes. In 2015, Pucher et al. highlighted a key challenge in HD classification: the prevalence of multiple symptoms among patients complicates the assessment of disease severity [43]. To address this issue, they developed the Sodergren severity scoring system, which evaluates itching, pain, and prolapse symptoms in patients with rectal bleeding, with a total score ranging from 0 to 14. This tool helps clinicians assess disease severity, track treatment outcomes, and monitor progression, recommending immediate surgery for scores of 6 or higher and rubber band ligation for scores below 6. Subsequently, Kuiper et al. introduced the Patient Reported Outcome Measure-Hemorrhoidal Impact and Satisfaction Score (PROM-HISS) which includes patient satisfaction as a third evaluation factor apart from symptoms and HR-QoL [40]. Although studies utilizing PROM-HISS have yet to be published, its design reflects a patient-centered approach that aligns with modern standards of outcome evaluation in proctology.

These tools collectively reflect the growing recognition of PROMs as essential instruments for evaluating treatment efficacy in HD. While PROMs are not designed for patient selection, their ability to capture symptom burden, patient perspectives, and HR-QoL provides a complementary approach to traditional classification systems. Incorporating PROMs into research and clinical practice is crucial for understanding the broader impact of treatments on patients’ lives and ensuring shared decision-making between clinicians and patients [45, 46].

However, we must note that while PROMs are highly useful for outcome evaluation, they are not designed for patient selection—a distinction that must be clearly drawn in research. The inclusion of the PROMs in this discussion is relevant only insofar as they offer an alternative means to assess disease impact post treatment.

## Conclusion

The Goligher classification system remains the most widely used method for grading HD in RCTs, but its limitations and inconsistent application highlight the need for more robust classification systems. Future research should aim to validate and refine alternative classification systems that account for both anatomical and symptomatic features, as well as integrate PROMs for a more comprehensive evaluation of treatment outcomes. Standardizing both patient selection criteria and outcome measurement tools will not only improve the quality of HD research but also enhance its comparability and clinical relevance.

## Supplementary Information

Below is the link to the electronic supplementary material.

Supplementary file1 (PDF 782 KB)

Supplementary file2 (DOCX 60 KB)

## Data Availability

The data that support the findings of this study are available from the corresponding authors upon reasonable request.
