# Supplementary material for: Limitations of the Goligher classification in randomized trials for hemorrhoidal disease: a qualitative systematic review of selection criteria
Source: Tech Coloproctol. 2025 Jun 10;29(1):133. doi: 10.1007/s10151-025-03170-y (PMC12152054; doi:10.1007/s10151-025-03170-y)
Supplement: Supplementary file 2 — Supplementary file2 (DOCX 60 KB) [file 10151_2025_3170_MOESM2_ESM.docx]

**SUPPLEMENTARY MATERIAL**

**Appendix 1: Search strategy**

**Initial search 14-12-2022:**

| Databases: |  |  |
| --- | --- | --- |
| PubMed, Embase (Ovid),  Cochrane CENTRAL, Web of Science | Before deduplication | After deduplication |
| Total | 6063 | 3377 |

**PUBMED**

1337 hits:

("Hemorrhoids"[Mesh] OR hemorrhoid*[tiab] OR haemorrhoid*[tiab])

AND

("Severity of Illness Index"[Mesh] OR "Symptom Assessment"[Mesh] OR "Patient Selection"[Mesh] OR "Patient Admission"[Mesh] OR "classification" [Subheading] OR classif*[tiab] OR score*[tiab] OR scoring*[tiab] OR algorithm*[tiab] OR grade*[tiab] OR grading[tiab] OR gradation*[tiab] OR occlusion*[tiab] OR symptom control*[tiab] OR symptom assess*[tiab] OR BPRST[tiab] OR patient select*[tiab] OR patient admission*[tiab] or patient recruitment*[tiab])

AND

("Surgical Procedures, Operative"[Mesh] OR "Anal Canal/surgery"[Mesh] OR haemorrhoidectom*[tiab] OR hemorrhoidectom*[tiab] OR hemorrhoidopex*[tiab] OR haemorrhoidopex*[tiab] OR surger*[tiab] OR surgical*[tiab] OR operation*[tiab] OR ligation*[tiab] OR rectal anal repair*[tiab] OR transanal hemorrhoidal dearterialization*[tiab] OR rubber band ligation*[tiab] OR RBL[tiab] OR sclerotherap*[tiab] OR aethoxysclero*[tiab] OR cryotherap*[tiab] OR infrared photo-coagulation*[tiab])

**EMBASE (OVID):**

Database(s): **Embase Classic+Embase**1947 to 2022 December 13
Search Strategy:

| **#** | **Searches** | **Results** |
| --- | --- | --- |
| 1 | hemorrhoid/ | 10953 |
| 2 | (hemorrhoid* or haemorrhoid*).ti,ab,kf. | 10919 |
| 3 | 1 or 2 | 14568 |
| 4 | "severity of illness index"/ or disease classification/ or classification/ or scoring system/ or disease severity/ or disease severity assessment/ or patient selection/ or hospital admission/ | 1771510 |
| 5 | (classif* or score* or scoring* or algorithm* or grade* or grading or gradation* or occlusion* or symptom control* or symptom assess* or BPRST or patient select* or patient admission* or patient recruitment*).ti,ab,kf. | 4145385 |
| 6 | 4 or 5 | 5238844 |
| 7 | hemorrhoid/su or anal canal/su or hemorrhoidectomy/ or anus surgery/ | 8380 |
| 8 | (haemorrhoidectom* or hemorrhoidectom* or hemorrhoidopex* or haemorrhoidopex* or surger* or surgical* or operation* or ligation* or rectal anal repair* or transanal hemorrhoidal dearterialization* or rubber band ligation* or RBL or sclerotherap* or aethoxysclero* or cryotherap* or infrared photo-coagulation*).ti,ab,kf. | 3703165 |
| 9 | 7 or 8 | 3705333 |
| 10 | 3 and 6 and 9 | 2511 |

[*Cochrane* Central Register of Controlled Trials](https://www.cochranelibrary.com/)

Issue 11 of 12, November 2022

ID Search Hits

#1 (hemorrhoid* or haemorrhoid*):ti,ab,kw 2034

#2 (classif* or score* or scoring* or algorithm* or grade* or grading or gradation* or occlusion* or symptom control* or symptom assess* or BPRST or patient select* or patient admission* or patient recruitment*):ti,ab,kw 524700

#3 MeSH descriptor: [Severity of Illness Index] explode all trees 21242

#4 MeSH descriptor: [Symptom Assessment] explode all trees 307

#5 #2 or #3 or #4 533852

#6 (haemorrhoidectom* or hemorrhoidectom* or hemorrhoidopex* or haemorrhoidopex* or surger* or surgical* or operation* or ligation* or rectal anal repair* or transanal hemorrhoidal dearterialization* or rubber band ligation* or RBL or sclerotherap* or aethoxysclero* or cryotherap* or infrared photo-coagulation*):ti,ab,kw 308531

#7 #1 and #5 and #6 in Trials 834

**Web of Science:**

1381 results:

((TS=(hemorrhoid* or haemorrhoid*)) AND TS=( classif* or score* or scoring* or algorithm* or grade* or grading or gradation* or occlusion* or symptom control* or symptom assess* or BPRST or patient select* or patient admission* or patient recruitment*)) AND TS=(haemorrhoidectom* or hemorrhoidectom* or hemorrhoidopex* or haemorrhoidopex* or surger* or surgical* or operation* or ligation* or rectal anal repair* or transanal hemorrhoidal dearterialization* or rubber band ligation* or RBL or sclerotherap* or aethoxysclero* or cryotherap* or infrared photo-coagulation*)

**Search update 19-10-2023:**

| Databases: |  |  |
| --- | --- | --- |
| PubMed, Embase (Ovid),  Cochrane CENTRAL, Web of Science | Before deduplication | After deduplication |
| Total | 629 | 227 |

**PUBMED**

92 hits:

("Hemorrhoids"[Mesh] OR hemorrhoid*[tiab] OR haemorrhoid*[tiab])

AND

("Severity of Illness Index"[Mesh] OR "Symptom Assessment"[Mesh] OR "Patient Selection"[Mesh] OR "Patient Admission"[Mesh] OR "classification" [Subheading] OR classif*[tiab] OR score*[tiab] OR scoring*[tiab] OR algorithm*[tiab] OR grade*[tiab] OR grading[tiab] OR gradation*[tiab] OR occlusion*[tiab] OR symptom control*[tiab] OR symptom assess*[tiab] OR BPRST[tiab] OR patient select*[tiab] OR patient admission*[tiab] or patient recruitment*[tiab])

AND

("Surgical Procedures, Operative"[Mesh] OR "Anal Canal/surgery"[Mesh] OR haemorrhoidectom*[tiab] OR hemorrhoidectom*[tiab] OR hemorrhoidopex*[tiab] OR haemorrhoidopex*[tiab] OR surger*[tiab] OR surgical*[tiab] OR operation*[tiab] OR ligation*[tiab] OR rectal anal repair*[tiab] OR transanal hemorrhoidal dearterialization*[tiab] OR rubber band ligation*[tiab] OR RBL[tiab] OR sclerotherap*[tiab] OR aethoxysclero*[tiab] OR cryotherap*[tiab] OR infrared photo-coagulation*[tiab])

AND

("2022/12/14"[Date - Publication] : "2023/10/19"[Date - Publication])

**EMBASE (OVID):**

Database(s): **Embase Classic+Embase**1947 to 2023 October 19
Search Strategy:

| **#** | **Searches** | **Results** |
| --- | --- | --- |
| 1 | hemorrhoid/ | 11566 |
| 2 | (hemorrhoid* or haemorrhoid*).ti,ab,kf. | 11445 |
| 3 | 1 or 2 | 15319 |
| 4 | "severity of illness index"/ or disease classification/ or classification/ or scoring system/ or disease severity/ or disease severity assessment/ or patient selection/ or hospital admission/ | 1867149 |
| 5 | (classif* or score* or scoring* or algorithm* or grade* or grading or gradation* or occlusion* or symptom control* or symptom assess* or BPRST or patient select* or patient admission* or patient recruitment*).ti,ab,kf. | 4432536 |
| 6 | 4 or 5 | 5585387 |
| 7 | hemorrhoid/su or anal canal/su or hemorrhoidectomy/ or anus surgery/ | 8696 |
| 8 | (haemorrhoidectom* or hemorrhoidectom* or hemorrhoidopex* or haemorrhoidopex* or surger* or surgical* or operation* or ligation* or rectal anal repair* or transanal hemorrhoidal dearterialization* or rubber band ligation* or RBL or sclerotherap* or aethoxysclero* or cryotherap* or infrared photo-coagulation*).ti,ab,kf. | 3886701 |
| 9 | 7 or 8 | 3888928 |
| 10 | 3 and 6 and 9 | 2671 |
| 11 | limit 10 to yr="2022 -Current" | 296 |

[***Cochrane* Central Register of Controlled Trials**](https://www.cochranelibrary.com/)

CENTRAL

Issue 10 of 12, October 2023

ID Search Hits

#1 (hemorrhoid* or haemorrhoid*):ti,ab,kw 2173

#2 (classif* or score* or scoring* or algorithm* or grade* or grading or gradation* or occlusion* or symptom control* or symptom assess* or BPRST or patient select* or patient admission* or patient recruitment*):ti,ab,kw 566588

#3 MeSH descriptor: [Severity of Illness Index] explode all trees 24290

#4 MeSH descriptor: [Symptom Assessment] explode all trees 502

#5 #2 or #3 or #4 576828

#6 (haemorrhoidectom* or hemorrhoidectom* or hemorrhoidopex* or haemorrhoidopex* or surger* or surgical* or operation* or ligation* or rectal anal repair* or transanal hemorrhoidal dearterialization* or rubber band ligation* or RBL or sclerotherap* or aethoxysclero* or cryotherap* or infrared photo-coagulation*):ti,ab,kw 333257

#7 #1 and #5 and #6 with Cochrane Library publication date Between Dec 2022 and Oct 2023, in Trials 63

**Web of Science**:

Publication years 2022-2023

178 results:

((TS=(hemorrhoid* or haemorrhoid*)) AND TS=( classif* or score* or scoring* or algorithm* or grade* or grading or gradation* or occlusion* or symptom control* or symptom assess* or BPRST or patient select* or patient admission* or patient recruitment*)) AND TS=(haemorrhoidectom* or hemorrhoidectom* or hemorrhoidopex* or haemorrhoidopex* or surger* or surgical* or operation* or ligation* or rectal anal repair* or transanal hemorrhoidal dearterialization* or rubber band ligation* or RBL or sclerotherap* or aethoxysclero* or cryotherap* or infrared photo-coagulation*)

**Appendix 2: Reference list of included studies in this review (n=162)**

1. Abd El-Wahab EH, Osman AG, Helmy M. Comparative study between pile plication technique and open hemorrhoidectomy in the management of noncomplicated second-degree and third-degree hemorrhoids. The Egyptian Journal of Surgery 41(4):p 1563-1571, October-December 2022. DOI: 10.4103/ejs.ejs_258_22
2. Abo-hashem AA, Sarhan A, Aly AM. Harmonic Scalpel compared with bipolar electro-cautery hemorrhoidectomy: a randomized controlled trial. Int J Surg. 2010;8(3):243-7. doi: 10.1016/j.ijsu.2010.01.010. Epub 2010 Feb 2. PMID: 20132916.
3. Ahmad A, Kalimuddin M, Sonkar AA, et al. A Randomized Clinical Study to Compare the Outcome of Hemorrhoidal Artery Ligation (HAL) Procedure with and without Doppler Guidance in Grades I–III Hemorrhoidal Disease. Indian J Surg 83, 1153–1157 (2021). https://doi.org/10.1007/s12262-020-02606-z
4. Ahmad A, Kant R, Gupta A. Comparative Analysis of Doppler Guided Hemorrhoidal Artery Ligation (DG-HAL) & Infrared Coagulation (IRC) in Management of Hemorrhoids. Indian J Surg. 2013 Aug;75(4):274-7. doi: 10.1007/s12262-012-0444-5. Epub 2012 May 6. PMID: 24426452; PMCID: PMC3726808.
5. Aigner F, Kronberger I, Oberwalder M, Loizides A, Ulmer H, Gruber L, Pratschke J, Peer S, Gruber H. Doppler-guided haemorrhoidal artery ligation with suture mucopexy compared with suture mucopexy alone for the treatment of Grade III haemorrhoids: a prospective randomized controlled trial. Colorectal Dis. 2016 Jul;18(7):710-6. doi: 10.1111/codi.13280. PMID: 26787597.
6. Alemrajabi M, Akbari A, Sohrabi S, Rezazadehkermani M, Moradi M, Agah S, Masoodi M. Simple mucopexy and hemorrhoidal arterial ligation with and without Doppler guide: a randomized clinical trial for short-term outcome. Ann Coloproctol. 2023 Aug;39(4):351-356. doi: 10.3393/ac.2022.00017.0002. Epub 2022 May 16. PMID: 35570403; PMCID: PMC10475797.
7. Alnajim AA, Al-Hakkak S, Muhammad ASAS, Al-Wadess AA, Ahmed MA. LigaSure or Diathermy Excision of III-IV Degree Pile? A Single-institution Experience: A Randomized Control Trial. Open Access Maced J Med Sci [Internet]. 2022 Apr. 14 [cited 2025 Jan. 26];10(B):1158-63. Available from: https://oamjms.eu/index.php/mjms/article/view/8878
8. Altomare DF, Milito G, Andreoli R, Arcanà F, Tricomi N, Salafia C, Segre D, Pecorella G, Pulvirenti d'Urso A, Cracco N, Giovanardi G, Romano G; Ligasure for Hemorrhoids Study Group. Ligasure Precise vs. conventional diathermy for Milligan-Morgan hemorrhoidectomy: a prospective, randomized, multicenter trial. Dis Colon Rectum. 2008 May;51(5):514-9. doi: 10.1007/s10350-007-9171-6. Epub 2008 Jan 30. PMID: 18231834.
9. Altomare DF, Pecorella G, Tegon G, Aquilino F, Pennisi D, De Fazio M. Does a more extensive mucosal excision prevent haemorrhoidal recurrence after stapled haemorrhoidopexy? Long-term outcome of a randomized controlled trial. Colorectal Dis. 2017 Jun;19(6):559-562. doi: 10.1111/codi.13549. PMID: 27801539.
10. Alvandipour M, Tavallaei M, Rezaei F, Khodabakhsh H. Postoperative outcomes of intrasphincteric botox injection during hemorrhoidectomy: A double-blind clinical trial. J Res Med Sci. 2021 Aug 30;26:53. doi: 10.4103/jrms.JRMS_612_18. PMID: 34729061; PMCID: PMC8506240.
11. Ammaturo C, Tufano A, Spiniello E, Sodano B, Iervolino EM, Brillantino A, Braccio B. Stapled haemorrhoidopexy vs. Milligan-Morgan haemorrhoidectomy for grade III haemorrhoids: a randomized clinical trial. G Chir. 2012 Oct;33(10):346-51. PMID: 23095566.
12. Andrews BT, Layer GT, Jackson BT, Nicholls RJ. Randomized trial comparing diathermy hemorrhoidectomy with the scissor dissection Milligan-Morgan operation. Dis Colon Rectum. 1993 Jun;36(6):580-3. doi: 10.1007/BF02049865. PMID: 8500376.
13. Arbman G, Krook H, Haapaniemi S. Closed vs. open hemorrhoidectomy--is there any difference? Dis Colon Rectum. 2000 Jan;43(1):31-4. doi: 10.1007/BF02237240. PMID: 10813120.
14. Armstrong DN, Ambroze WL, Schertzer ME, Orangio GR. Harmonic Scalpel vs. electrocautery hemorrhoidectomy: a prospective evaluation. Dis Colon Rectum. 2001 Apr;44(4):558-64. doi: 10.1007/BF02234329. PMID: 11330583.
15. Arroyo A, Pérez F, Miranda E, Serrano P, Candela F, Lacueva J, Hernández H, Calpena R. Open versus closed day-case haemorrhoidectomy: is there any difference? Results of a prospective randomised study. Int J Colorectal Dis. 2004 Jul;19(4):370-3. doi: 10.1007/s00384-003-0573-1. Epub 2004 Mar 25. PMID: 15170517.
16. Arslani N, Patrlj L, Rajković Z, Papeš D, Altarac S. A randomized clinical trial comparing Ligasure versus stapled hemorrhoidectomy. Surg Laparosc Endosc Percutan Tech. 2012 Feb;22(1):58-61. doi: 10.1097/SLE.0b013e318247d966. PMID: 22318061.
17. Baig AA, Mehmood MS, Khalid R, Ghufran S, Chaudhry SM, Mehbub H. Comparative Study between Milligan Morgan Versus Ligasure Haemorrhoidectomy. Pakistan Journal of Medical & Health Sciences. 2022; 16(11), 235. https://doi.org/10.53350/pjmhs20221611235
18. Bakhtiar N, Moosa FA, Jaleel F, Qureshi NA, Jawaid M. Comparison of hemorrhoidectomy by LigaSure with conventional Milligan Morgan's hemorrhoidectomy. Pak J Med Sci. 2016 May-Jun;32(3):657-61. doi: 10.12669/pjms.323.9976. PMID: 27375709; PMCID: PMC4928418.
19. Basdanis G, Papadopoulos VN, Michalopoulos A, Apostolidis S, Harlaftis N. Randomized clinical trial of stapled hemorrhoidectomy vs open with Ligasure for prolapsed piles. Surg Endosc. 2005 Feb;19(2):235-9. doi: 10.1007/s00464-004-9098-0. Epub 2004 Dec 2. PMID: 15573239.
20. Bessa SS. Ligasure vs. conventional diathermy in excisional hemorrhoidectomy: a prospective, randomized study. Dis Colon Rectum. 2008 Jun;51(6):940-4. doi: 10.1007/s10350-008-9214-7. Epub 2008 Feb 14. PMID: 18273670.
21. Bessa SS. Diathermy excisional hemorrhoidectomy: a prospective randomized study comparing pedicle ligation and pedicle coagulation. Dis Colon Rectum. 2011 Nov;54(11):1405-11. doi: 10.1097/DCR.0b013e318222b5a9. PMID: 21979186.
22. Bikhchandani J, Agarwal PN, Kant R, Malik VK. Randomized controlled trial to compare the early and mid-term results of stapled versus open hemorrhoidectomy. Am J Surg. 2005 Jan;189(1):56-60. doi: 10.1016/j.amjsurg.2004.03.014. PMID: 15701493.
23. Bilgin Y, Hot S, Barlas İS, Akan A, Eryavuz Y. Short- and long-term results of harmonic scalpel hemorrhoidectomy versus stapler hemorrhoidopexy in treatment of hemorrhoidal disease. Asian J Surg. 2015 Oct;38(4):214-9. doi: 10.1016/j.asjsur.2014.09.004. Epub 2014 Nov 4. PMID: 25451631.
24. Boccasanta P, Capretti PG, Venturi M, Cioffi U, De Simone M, Salamina G, Contessini-Avesani E, Peracchia A. Randomised controlled trial between stapled circumferential mucosectomy and conventional circular hemorrhoidectomy in advanced hemorrhoids with external mucosal prolapse. Am J Surg. 2001 Jul;182(1):64-8. doi: 10.1016/s0002-9610(01)00654-7. PMID: 11532418.
25. Brown SR, Tiernan JP, Watson AJM, Biggs K, Shephard N, Wailoo AJ, Bradburn M, Alshreef A, Hind D; HubBLe Study team. Haemorrhoidal artery ligation versus rubber band ligation for the management of symptomatic second-degree and third-degree haemorrhoids (HubBLe): a multicentre, open-label, randomised controlled trial. Lancet. 2016 Jul 23;388(10042):356-364. doi: 10.1016/S0140-6736(16)30584-0. Epub 2016 May 25. Erratum in: Lancet. 2016 Jul 23;388(10042):342. doi: 10.1016/S0140-6736(16)31108-4. PMID: 27236344; PMCID: PMC4956910.
26. Bulus H, Tas A, Coskun A, Kucukazman M. Evaluation of two hemorrhoidectomy techniques: harmonic scalpel and Ferguson's with electrocautery. Asian J Surg. 2014 Jan;37(1):20-3. doi: 10.1016/j.asjsur.2013.04.002. Epub 2013 May 28. PMID: 23726831.
27. Bursics A, Morvay K, Kupcsulik P, Flautner L. Comparison of early and 1-year follow-up results of conventional hemorrhoidectomy and hemorrhoid artery ligation: a randomized study. Int J Colorectal Dis. 2004 Mar;19(2):176-80. doi: 10.1007/s00384-003-0517-9. Epub 2003 Jul 5. PMID: 12845454.
28. Carvajal López F, Hoyuela Alonso C, Juvany Gómez M, Troyano Escribano D, Trias Bisbal MA, Martrat Macià A, Ardid Brito J. Prospective Randomized Trial Comparing HAL-RAR Versus Excisional Hemorrhoidectomy: Postoperative Pain, Clinical Outcomes, and Quality of Life. Surg Innov. 2019 Jun;26(3):328-336. doi: 10.1177/1553350618822644. Epub 2019 Jan 9. PMID: 30621513.
29. Cemil A, Ugur K, Salih GM, Merve K, Guray DM, Emine BS. Comparison of Laser Hemorrhoidoplasty and Milligan-Morgan Hemorrhoidectomy Techniques in the Treatment of Grade 2 and 3 Hemorrhoidal Disease. Am Surg. 2024 Apr;90(4):662-671. doi: 10.1177/00031348231207301. Epub 2023 Oct 17. PMID: 37846728.
30. Cheetham MJ, Cohen CR, Kamm MA, Phillips RK. A randomized, controlled trial of diathermy hemorrhoidectomy vs. stapled hemorrhoidectomy in an intended day-care setting with longer-term follow-up. Dis Colon Rectum. 2003 Apr;46(4):491-7. doi: 10.1007/s10350-004-6588-z. PMID: 12682543.
31. Chung CC, Ha JP, Tai YP, Tsang WW, Li MK. Double-blind, randomized trial comparing Harmonic Scalpel hemorrhoidectomy, bipolar scissors hemorrhoidectomy, and scissors excision: ligation technique. Dis Colon Rectum. 2002 Jun;45(6):789-94. doi: 10.1007/s10350-004-6299-5. PMID: 12072632.
32. Chung YC, Wu HJ. Clinical experience of sutureless closed hemorrhoidectomy with LigaSure. Dis Colon Rectum. 2003 Jan;46(1):87-92. doi: 10.1007/s10350-004-6501-9. PMID: 12544527.
33. Chung CC, Cheung HY, Chan ES, Kwok SY, Li MK. Stapled hemorrhoidopexy vs. Harmonic Scalpel hemorrhoidectomy: a randomized trial. Dis Colon Rectum. 2005 Jun;48(6):1213-9. doi: 10.1007/s10350-004-0918-z. PMID: 15793648.
34. Correa-Rovelo JM, Tellez O, Obregón L, Miranda-Gomez A, Moran S. Stapled rectal mucosectomy vs. closed hemorrhoidectomy: a randomized, clinical trial. Dis Colon Rectum. 2002 Oct;45(10):1367-74; discussion 1374-5. doi: 10.1007/s10350-004-6426-3. PMID: 12394436.
35. Denoya PI, Fakhoury M, Chang K, Fakhoury J, Bergamaschi R. Dearterialization with mucopexy versus haemorrhoidectomy for grade III or IV haemorrhoids: short-term results of a double-blind randomized controlled trial. Colorectal Dis. 2013;15(10):1281-8. doi: 10.1111/codi.12303. PMID: 23711288.
36. Elmér SE, Nygren JO, Lenander CE. A randomized trial of transanal hemorrhoidal dearterialization with anopexy compared with open hemorrhoidectomy in the treatment of hemorrhoids. Dis Colon Rectum. 2013 Apr;56(4):484-90. doi: 10.1097/DCR.0b013e31827a8567. PMID: 23478616.
37. Elshazly WG, Gazal AE, Madbouly K, Hussen A. Ligation anopexy versus hemorrhoidectomy in the treatment of second- and third-degree hemorrhoids. Tech Coloproctol. 2015 Jan;19(1):29-34. doi: 10.1007/s10151-014-1235-8. Epub 2014 Nov 25. PMID: 25421703.
38. Elshazly WG, Abo Elros MA, Ali AS, Radwan AM. Randomized Controlled Trial to Compare Stapled Hemorrhoidopexy Plus Ligation Anopexy With Stapled Hemorrhoidopexy for Managing Grade III and IV Hemorrhoidal Disease. Dis Colon Rectum. 2024 Jun 1;67(6):812-819. doi: 10.1097/DCR.0000000000003273. Epub 2024 Feb 21. PMID: 38380816.
39. Eskandaros, Moheb S.; Darwish, Ahmed A.. Comparative study between Milligan-Morgan hemorrhoidectomy, stapled hemorrhoidopexy, and laser hemorrhoidoplasty in patients with third degree hemorrhoids: a prospective study. The Egyptian Journal of Surgery 39(2):p 352-363, Apr–Jun 2020. | DOI: 10.4103/ejs.ejs_214_19
40. Falsarella PM, Nasser F, Affonso BB, Galastri FL, Motta-Leal-Filho JMD, Valle LGM, Cunha MJS, Araújo SEA, Garcia RG, Katz M. Embolization of the Superior Rectal Arteries versus Closed Hemorrhoidectomy (Ferguson Technique) in the Treatment of Hemorrhoidal Disease: A Randomized Clinical Trial. J Vasc Interv Radiol. 2023 May;34(5):736-744.e1. doi: 10.1016/j.jvir.2023.01.022. Epub 2023 Jan 31. PMID: 36736690.
41. Fang Y, Zhang Y, Zhang D, Zhao Q, Li L. Clinical observation on treatment of mixed hemorrhoids with milligan morgan hemorrhoidectomy combined with purse-string suture. Int J Clin Exp Med 2018;11(11):12555-12562. ISNN1940-5901/IJCEM0080094
42. Fareed M, El-Awady S, Abd-El monaem H, Aly A. Randomized trial comparing LigaSure to closed Ferguson hemorrhoidectomy. Tech Coloproctol. 2009 Sep;13(3):243-6. doi: 10.1007/s10151-009-0520-4. Epub 2009 Jul 24. PMID: 19629378.
43. Festen S, van Hoogstraten MJ, van Geloven AA, Gerhards MF. Treatment of grade III and IV haemorrhoidal disease with PPH or THD. A randomized trial on postoperative complications and short-term results. Int J Colorectal Dis. 2009 Dec;24(12):1401-5. doi: 10.1007/s00384-009-0803-2. PMID: 19798507.
44. Filingeri V, Gravante G, Baldessari E, Grimaldi M, Casciani CU. Prospective randomized trial of submucosal hemorrhoidectomy with radiofrequency bistoury vs. conventional Parks' operation. Tech Coloproctol. 2004 Mar;8(1):31-6. doi: 10.1007/s10151-004-0048-6. PMID: 15057587.
45. Franklin EJ, Seetharam S, Lowney J, Horgan PG. Randomized, clinical trial of Ligasure vs conventional diathermy in hemorrhoidectomy. Dis Colon Rectum. 2003 Oct;46(10):1380-3. doi: 10.1007/s10350-004-6754-3. PMID: 14530679.
46. Ganio E, Altomare DF, Gabrielli F, Milito G, Canuti S. Prospective randomized multicentre trial comparing stapled with open haemorrhoidectomy. Br J Surg. 2001 May;88(5):669-74. doi: 10.1046/j.0007-1323.2001.01772.x. PMID: 11350437.
47. Genova P, Damiano G, Lo Monte AI, Genova G. Transanal hemorrhoidal dearterialization versus Milligan-Morgan hemorrhoidectomy in grade III/IV hemorrhoids. Ann Ital Chir. 2019;90:145-151. PMID: 31182699.
48. Gentile M, De Rosa M, Carbone G, Pilone V, Mosella F, Forestieri P. LigaSure Haemorrhoidectomy versus Conventional Diathermy for IV-Degree Haemorrhoids: Is It the Treatment of Choice? A Randomized, Clinical Trial. ISRN Gastroenterol. 2011;2011:467258. doi: 10.5402/2011/467258. Epub 2010 Nov 21. PMID: 21991510; PMCID: PMC3168454.
49. Gerjy R, Lindhoff-Larson A, Nyström PO. Grade of prolapse and symptoms of haemorrhoids are poorly correlated: result of a classification algorithm in 270 patients. Colorectal Dis. 2008 Sep;10(7):694-700. doi: 10.1111/j.1463-1318.2008.01498.x. Epub 2008 Feb 21. PMID: 18294262.
50. Giarratano G, Toscana E, Toscana C, Petrella G, Shalaby M, Sileri P. Transanal Hemorrhoidal Dearterialization Versus Stapled Hemorrhoidopexy: Long-Term Follow-up of a Prospective Randomized Study. Surg Innov. 2018 Jun;25(3):236-241. doi: 10.1177/1553350618761757. Epub 2018 Mar 5. PMID: 29504471.
51. Giordano P, Nastro P, Davies A, Gravante G. Prospective evaluation of stapled haemorrhoidopexy versus transanal haemorrhoidal dearterialisation for stage II and III haemorrhoids: three-year outcomes. Tech Coloproctol. 2011 Mar;15(1):67-73. doi: 10.1007/s10151-010-0667-z. Epub 2011 Feb 12. PMID: 21318581; PMCID: PMC3046344.
52. Gravié JF, Lehur PA, Huten N, Papillon M, Fantoli M, Descottes B, Pessaux P, Arnaud JP. Stapled hemorrhoidopexy versus milligan-morgan hemorrhoidectomy: a prospective, randomized, multicenter trial with 2-year postoperative follow up. Ann Surg. 2005 Jul;242(1):29-35. doi: 10.1097/01.sla.0000169570.64579.31. PMID: 15973098; PMCID: PMC1357701.
53. Gupta PJ. A comparative study between radiofrequency ablation with plication and Milligan-Morgan hemorrhoidectomy for grade III hemorrhoids. Tech Coloproctol. 2004 Nov;8(3):163-8. doi: 10.1007/s10151-004-0081-5. PMID: 15654523.
54. Gupta PJ, Heda PS, Kalaskar S. Radiofrequency ablation and plication--a new technique for prolapsing hemorrhoidal disease. Curr Surg. 2006 Jan-Feb;63(1):44-50. doi: 10.1016/j.cursur.2005.04.016. PMID: 16373160.
55. Hetzer FH, Demartines N, Handschin AE, Clavien PA. Stapled vs excision hemorrhoidectomy: long-term results of a prospective randomized trial. Arch Surg. 2002 Mar;137(3):337-40. doi: 10.1001/archsurg.137.3.337. PMID: 11888463.
56. Hidalgo-Grau LA, Piedrafita-Serra E, Ruiz-Edo N, Llorca-Cardeñosa S, Heredia-Budó A, Estrada-Ferrer O, Suñol-Sala X. Prospective Randomized Study on Stapled Anopexy Height and Its Influence on Recurrence for Hemorrhoidal Disease Treatment. World J Surg. 2020 Nov;44(11):3936-3942. doi: 10.1007/s00268-020-05676-y. PMID: 32647985.
57. Ho KS, Ho YH. Prospective randomized trial comparing stapled hemorrhoidopexy versus closed Ferguson hemorrhoidectomy. Tech Coloproctol. 2006 Oct;10(3):193-7. doi: 10.1007/s10151-006-0279-9. Epub 2006 Sep 20. PMID: 16969617; PMCID: PMC2779391.
58. Ho YH, Cheong WK, Tsang C, Ho J, Eu KW, Tang CL, Seow-Choen F. Stapled hemorrhoidectomy--cost and effectiveness. Randomized, controlled trial including incontinence scoring, anorectal manometry, and endoanal ultrasound assessments at up to three months. Dis Colon Rectum. 2000 Dec;43(12):1666-75. doi: 10.1007/BF02236847. PMID: 11156449.
59. Huang WS, Chin CC, Yeh CH, Lin PY, Wang JY. Randomized comparison between stapled hemorrhoidopexy and Ferguson hemorrhoidectomy for grade III hemorrhoids in Taiwan: a prospective study. Int J Colorectal Dis. 2007 Aug;22(8):955-61. doi: 10.1007/s00384-006-0244-0. Epub 2006 Dec 14. PMID: 17171354.
60. Huang H, Gu Y, Ji L, Li Y, Xu S, Guo T, Xu M. A NEW MIXED SURGICAL TREATMENT FOR GRADES III AND IV HEMORRHOIDS: MODIFIED SELECTIVE HEMORRHOIDECTOMY COMBINED WITH COMPLETE ANAL EPITHELIAL RETENTION. Arq Bras Cir Dig. 2021 Oct 15;34(2):e1594. doi: 10.1590/0102-672020210002e1594. PMID: 34669884; PMCID: PMC8521818.
61. Huang H, Tao L, Jiang J, Wei J, Ji L. Tissue-selecting-technique mega-window stapler combined with anal canal epithelial preservation operation in prolapsed hemorrhoids. Asian J Surg. 2023 Feb;46(2):807-815. doi: 10.1016/j.asjsur.2022.07.149. Epub 2022 Aug 10. PMID: 35961908.
62. Huang H, Wen K, Ding X, Yan L, Gu Y, Ji L. The efficiency and safety of modified tissue-selecting therapy stapler combined with complete anal canal epithelial preservation operation in circumferential mixed hemorrhoids: a randomized controlled trial. Langenbecks Arch Surg. 2023 Aug 24;408(1):332. doi: 10.1007/s00423-023-03081-2. PMID: 37620667.
63. Infantino A, Altomare DF, Bottini C, Bonanno M, Mancini S; THD group of the SICCR (Italian Society of Colorectal Surgery); Yalti T, Giamundo P, Hoch J, El Gaddal A, Pagano C. Prospective randomized multicentre study comparing stapler haemorrhoidopexy with Doppler-guided transanal haemorrhoid dearterialization for third-degree haemorrhoids. Colorectal Dis. 2012 Feb;14(2):205-11. doi: 10.1111/j.1463-1318.2011.02628.x. PMID: 21689317.
64. Irfan F, Salim M, Abid KJ. Outcome of stapled haemorrhoidectomy versus open haemorrhoidectomy: A randomized control trial. Pakinstan Journal of Medical & Health Sciences. 2014;8(2):491-495.
65. Izadpanah A, Hosseini S, Mahjoob M. Comparison of electrotherapy, rubber band ligation and hemorrhoidectomy in the treatment of hemorrhoids: a clinical and manometric study. Middle East J Dig Dis. 2010 Jan;2(1):9-13. PMID: 25197506; PMCID: PMC4154911.
66. Javed S, Kaiser A, Khan AZ, Javed A, Chaudhary S, Javed A, Shahid MH. ENSEAL® Hemorrhoidectomy, a Novel Technique, Versus Conventional Open Method for the Management of Grade III and IV Hemorrhoids. Cureus. 2022 Oct 29;14(10):e30834. doi: 10.7759/cureus.30834. PMID: 36407175; PMCID: PMC9661451.
67. Jayne DG, Botterill I, Ambrose NS, Brennan TG, Guillou PJ, O'Riordain DS. Randomized clinical trial of Ligasure versus conventional diathermy for day-case haemorrhoidectomy. Br J Surg. 2002 Apr;89(4):428-32. doi: 10.1046/j.0007-1323.2002.02056.x. PMID: 11952582.
68. Jia XQ, Cao WW, Quan LF, Zhao WB, Cheng F, Jia S, Feng LQ, Wei XF, Xie ZN, Wang D, Xu CY, Cui CH, Cai XJ, He LY, Wang ZJ, Tian Y, Shi SM, Sun SM, Su L, Zhai MF. Effect of High Suspension and Low Incision Surgery Based on Traditional Ligation of Chinese Medicine in Treatment of Mixed Haemorrhoids: A Multi-centre, Randomized, Single-Blind, Non-inferiority Clinical Trial. Chin J Integr Med. 2021 Sep;27(9):649-655. doi: 10.1007/s11655-021-3329-2. Epub 2021 Mar 12. PMID: 33709237.
69. Jin L, Yang H, Qin K, Li Y, Cui C, Wu R, Wang Z, Wu J. Efficacy of modified rubber band ligation in the treatment of grade III internal hemorrhoids. Ann Palliat Med. 2021 Feb;10(2):1191-1197. doi: 10.21037/apm-19-657. Epub 2020 Oct 12. PMID: 33081476.
70. Jóhannsson HO, Påhlman L, Graf W. Randomized clinical trial of the effects on anal function of Milligan-Morgan versus Ferguson haemorrhoidectomy. Br J Surg. 2006 Oct;93(10):1208-14. doi: 10.1002/bjs.5408. PMID: 16952213.
71. Jyoti K, Sharma A, Rao PP. Randomised Clinical Trial of Harmonic Scalpel Haemorrhoidectomy Versus Stapled Haemorrhoidopexy in grade III / IV Haemorrhoids. Indian J Surg. 2004;86:540–543. https://doi.org/10.1007/s12262-023-03901-1
72. Kairaluoma M, Nuorva K, Kellokumpu I. Day-case stapled (circular) vs. diathermy hemorrhoidectomy: a randomized, controlled trial evaluating surgical and functional outcome. Dis Colon Rectum. 2003 Jan;46(1):93-9. doi: 10.1007/s10350-004-6502-8. PMID: 12544528.
73. Kasthuri D, Chandrasekhara Reddy E. Harmonic Scalpel Haemorrhoidectomy vs. Conventional Milligan and Morgan Haemorrhoidectomy: A Prospective Study. Journal of Evolution of Medical and Dental Science. 2015 Sep;4(73):12630-12638, DOI: 10.14260/jemds/2015/1821
74. Khalil KH, O'Bichere A, Sellu D. Randomized clinical trial of sutured versus stapled closed haemorrhoidectomy. Br J Surg. 2000 Oct;87(10):1352-5. doi: 10.1046/j.1365-2168.2000.01624.x. PMID: 11044160.
75. Khan S, Pawlak SE, Eggenberger JC, Lee CS, Szilagy EJ, Wu JS, Margolin M D DA. Surgical treatment of hemorrhoids: prospective, randomized trial comparing closed excisional hemorrhoidectomy and the Harmonic Scalpel technique of excisional hemorrhoidectomy. Dis Colon Rectum. 2001 Jun;44(6):845-9. doi: 10.1007/BF02234706. PMID: 11391146.
76. Khan Z, Razzaq S, Zareen N, Hussain A, Kashif M, Khan JI. Conventional Hemorrhoidectomy Versus Stapled Hemorrhoidopexy: Compare the Outcomes of both Techniques in Patients With Grade III and IV Hemorrhoids. P J M H S. 2020 Apr-Jun;2:588-591.
77. Khanna R, Khanna S, Bhadani S, Singh S, Khanna AK. Comparison of Ligasure Hemorrhoidectomy with Conventional Ferguson's Hemorrhoidectomy. Indian J Surg. 2010 Aug;72(4):294-7. doi: 10.1007/s12262-010-0192-3. Epub 2010 Nov 18. PMID: 21938191; PMCID: PMC3002768.
78. Kim JS, Vashist YK, Thieltges S, Zehler O, Gawad KA, Yekebas EF, Izbicki JR, Kutup A. Stapled hemorrhoidopexy versus Milligan-Morgan hemorrhoidectomy in circumferential third-degree hemorrhoids: long-term results of a randomized controlled trial. J Gastrointest Surg. 2013 Jul;17(7):1292-8. doi: 10.1007/s11605-013-2220-7. Epub 2013 May 14. PMID: 23670518.
79. Kraemer M, Parulava T, Roblick M, Duschka L, Müller-Lobeck H. Prospective, randomized study: proximate PPH stapler vs. LigaSure for hemorrhoidal surgery. Dis Colon Rectum. 2005 Aug;48(8):1517-22. doi: 10.1007/s10350-005-0067-z. PMID: 15937619.
80. Krska Z, Kvasnièka J, Faltýn J, Schmidt D, Sváb J, Kormanová K, Hubík J. Surgical treatment of haemorrhoids according to Longo and Milligan Morgan: an evaluation of postoperative tissue response. Colorectal Dis. 2003 Nov;5(6):573-6. doi: 10.1046/j.1463-1318.2003.00551.x. PMID: 14617243.
81. Kwok SY, Chung CC, Tsui KK, Li MK. A double-blind, randomized trial comparing Ligasure and Harmonic Scalpel hemorrhoidectomy. Dis Colon Rectum. 2005 Feb;48(2):344-8. doi: 10.1007/s10350-004-0845-z. PMID: 15616753.
82. Lau PY, Meng WC, Yip AW. Stapled haemorrhoidectomy in Chinese patients: a prospective randomised control study. Hong Kong Med J. 2004 Dec;10(6):373-7. PMID: 15591594.
83. Lehur PA, Didnée AS, Faucheron JL, Meurette G, Zerbib P, Siproudhis L, Vinson-Bonnet B, Dubois A, Casa C, Hardouin JB, Durand-Zaleski I; LigaLongo Study Group. Cost-effectiveness of New Surgical Treatments for Hemorrhoidal Disease: A Multicentre Randomized Controlled Trial Comparing Transanal Doppler-guided Hemorrhoidal Artery Ligation With Mucopexy and Circular Stapled Hemorrhoidopexy. Ann Surg. 2016 Nov;264(5):710-716. doi: 10.1097/SLA.0000000000001770. PMID: 27741005.
84. Leung ALH, Cheung TPP, Tung K, Tsang YP, Cheung H, Lau CW, Tang CN. A prospective randomized controlled trial evaluating the short-term outcomes of transanal hemorrhoidal dearterialization versus tissue-selecting technique. Tech Coloproctol. 2017 Sep;21(9):737-743. doi: 10.1007/s10151-017-1669-x. Epub 2017 Sep 20. PMID: 28932913.
85. Lim SY, Rajandram R, Roslani AC. Comparison of post-operative bleeding incidence in laser hemorrhoidoplasty with and without hemorrhoidal artery ligation: a double-blinded randomized controlled trial. BMC Surg. 2022 Apr 21;22(1):146. doi: 10.1186/s12893-022-01594-z. PMID: 35449097; PMCID: PMC9022276.
86. Lin HC, He QL, Shao WJ, Chen XL, Peng H, Xie SK, Wang XX, Ren DL. Partial Stapled Hemorrhoidopexy Versus Circumferential Stapled Hemorrhoidopexy for Grade III to IV Prolapsing Hemorrhoids: A Randomized, Noninferiority Trial. Dis Colon Rectum. 2019 Feb;62(2):223-233. doi: 10.1097/DCR.0000000000001261. PMID: 30489326; PMCID: PMC6365260.
87. Lucarelli P, Picchio M, Caporossi M, De Angelis F, Di Filippo A, Stipa F, Spaziani E. Transanal haemorrhoidal dearterialisation with mucopexy versus stapler haemorrhoidopexy: a randomised trial with long-term follow-up. Ann R Coll Surg Engl. 2013 May;95(4):246-51. doi: 10.1308/003588413X13511609958136. Retraction in: Ann R Coll Surg Engl. 2014 Mar;96(2):94. doi: 10.1308/rcsann.2014.94. PMID: 23676807; PMCID: PMC4132497.
88. Marcet J. Ferrara A, Rivadeneira DE, Erbella J, Papaconstantinou HT. Prospective, Multicenter Randomized Controlled Trial Comparing Two Hemorrhoidopexy Staplers: The HEMOSTASIS Study. International Surgery. 2018;103(3-4):129-138.
89. Mathai V, Ong BC, Ho YH. Randomized controlled trial of lateral internal sphincterotomy with haemorrhoidectomy. BJS. 1996 Mar;83(3):380–382. doi: 10.1002/bjs.1800830327.
90. Mehigan BJ, Monson JR, Hartley JE. Stapling procedure for haemorrhoids versus Milligan-Morgan haemorrhoidectomy: randomised controlled trial. Lancet. 2000 Mar 4;355(9206):782-5. doi: 10.1016/S0140-6736(99)08362-2. PMID: 10711925.
91. Mengal MA, Qasim KF, Baloch FA, Elahi SAB. Early outcomes of stapled vs conventional hemorrhoidectomy. P J M H S. 2017 Jan-Mar;11(1):180-183.
92. Mert T. Comparison of Laser Haemorrhoidoplasty and Ferguson Haemorrhoidectomy in Treating Grade III and Grade IV Haemorrhoids: A Prospective Randomised Study. JCPSP. 2023 Jan. doi: 10.29271/jcpsp.2023.01.45
93. Milito G, Gargiani M, Cortese F. Randomised trial comparing LigaSure haemorrhoidectomy with the diathermy dissection operation. Tech Coloproctol. 2002 Dec;6(3):171-5. doi: 10.1007/s101510200038. PMID: 12525911.
94. Mohamed KMS, El-Fattah MQA, Elbarbary MG. Doppler-guided hemorrhoidal artery ligation versus digital- guided ligation in management of second-degree and third- degree hemorrhoids. The Egyptian Journal of Surgery. 2023;41:1722-1729. DOI: 10.4103/ejs.ejs_259_22
95. Murie JA, Mackenzie I, Sim AJ. Comparison of rubber band ligation and haemorrhoidectomy for second- and third-degree haemorrhoids: a prospective clinical trial. Br J Surg. 1980 Nov;67(11):786-8. doi: 10.1002/bjs.1800671108. PMID: 6968608.
96. Muzi MG, Milito G, Nigro C, Cadeddu F, Andreoli F, Amabile D, Farinon AM. Randomized clinical trial of LigaSure and conventional diathermy haemorrhoidectomy. Br J Surg. 2007 Aug;94(8):937-42. doi: 10.1002/bjs.5904. PMID: 17636512.
97. Nada MAM, Awad PBA, Kirollos AMA, Abdelaziz MM, Mohamed KMS, Awad KBA, Hassan BHA. Comparison between stapled hemorrhoidopexy and harmonic scalpel hemorrhoidectomy in the management of third- and fourth-degree piles: a randomized clinical trial. Chirurgie (Heidelb). 2024 Dec;95(Suppl 1):14-22. doi: 10.1007/s00104-023-02010-9. Epub 2023 Dec 29. PMID: 38157069; PMCID: PMC11649745.
98. Naderan M, Shoar S, Nazari M, Elsayed A, Mahmoodzadeh H, Khorgami Z. A Randomized Controlled Trial Comparing Laser Intra-Hemorrhoidal Coagulation and Milligan-Morgan Hemorrhoidectomy. J Invest Surg. 2017 Oct;30(5):325-331. doi: 10.1080/08941939.2016.1248304. Epub 2016 Nov 2. PMID: 27806213.
99. Naresh Kumar S, Davender, Raj GS. A Study on Safety and Efficacy of Haemorrhoidectomy (Milligan Morgan Vs Stapler Haemorrhoidopexy). Int J of Toxicological and Pharmacological Research. 2023;13(7):23-29.
100. Naz S, Khan U, Jan H, Khan NS, Shahzad T, Farooq M. Comparison of haemorrhoidectomy using ligasure with open (Milligan Morgan haemorrhoidectomy). P J M H S. 2022 Apr;16(4):84-85. DOI: https://doi.org/10.53350/pjmhs2216484
101. Neves S, Falcão D, Povo A, Castro-Poças F, Oliveira J, Salgueiro P. 3% polidocanol foam sclerotherapy versus hemorrhoidal artery ligation with recto anal repair in hemorrhoidal disease grades II-III: a randomized, pilot trial. Rev Esp Enferm Dig. 2023 Mar;115(3):115-120. doi: 10.17235/reed.2022.8568/2022. PMID: 35638762.
102. Nikshoar MR, Maleki Z, Nemati Honar B. The Clinical Efficacy of Infrared Photocoagulation Versus Closed Hemorrhoidectomy in Treatment of Hemorrhoid. J Lasers Med Sci. 2018 Winter;9(1):23-26. doi: 10.15171/jlms.2018.06. Epub 2017 Dec 26. PMID: 29399307; PMCID: PMC5775951.
103. Nyström PO, Qvist N, Raahave D, Lindsey I, Mortensen N; Stapled or Open Pile Procedure (STOPP) trial study group. Randomized clinical trial of symptom control after stapled anopexy or diathermy excision for haemorrhoid prolapse. Br J Surg. 2010 Feb;97(2):167-76. doi: 10.1002/bjs.6804. PMID: 20035531.
104. Onur Gülseren M, Dinc T, Özer V, Yildiz B, Cete M, Coskun F. Randomized Controlled Trial Comparing the Effects of Vessel Sealing Device and Milligan Morgan Technique on Postoperative Pain Perception after Hemorrhoidectomy. Dig Surg. 2015;32(4):258-61. doi: 10.1159/000381754. Epub 2015 May 21. PMID: 26022195.
105. Ortiz H, Marzo J, Armendariz P. Randomized clinical trial of stapled haemorrhoidopexy versus conventional diathermy haemorrhoidectomy. Br J Surg. 2002 Nov;89(11):1376-81. doi: 10.1046/j.1365-2168.2002.02237.x. PMID: 12390376.
106. Ortiz H, Marzo J, Armendáriz P, De Miguel M. Stapled hemorrhoidopexy vs. diathermy excision for fourth-degree hemorrhoids: a randomized, clinical trial and review of the literature. Dis Colon Rectum. 2005 Apr;48(4):809-15. doi: 10.1007/s10350-004-0861-z. PMID: 15785901.
107. Palazzo FF, Francis DL, Clifton MA. Randomized clinical trial of Ligasure versus open haemorrhoidectomy. Br J Surg. 2002 Feb;89(2):154-7. doi: 10.1046/j.0007-1323.2001.01993.x. PMID: 11856126.
108. Palimento D, Picchio M, Attanasio U, Lombardi A, Bambini C, Renda A. Stapled and open hemorrhoidectomy: randomized controlled trial of early results. World J Surg. 2003 Feb;27(2):203-7. doi: 10.1007/s00268-002-6459-5. PMID: 12616437.
109. Pandini LC, Nahas SC, Nahas CS, Marques CF, Sobrado CW, Kiss DR. Surgical treatment of haemorrhoidal disease with CO2 laser and Milligan-Morgan cold scalpel technique. Colorectal Dis. 2006 Sep;8(7):592-5. doi: 10.1111/j.1463-1318.2006.01023.x. PMID: 16919112.
110. Pattana-Arun J, Sooriprasoet N, Sahakijrungruang C, Tantiphlachiva K, Rojanasakul A. Closed vs ligasure hemorrhoidectomy: a prospective, randomized clinical trial. J Med Assoc Thai. 2006 Apr;89(4):453-8. PMID: 16696389.
111. Pavlidis T, Papaziogas B, Souparis A, Patsas A, Koutelidakis I, Papaziogas T. Modern stapled Longo procedure vs. conventional Milligan-Morgan hemorrhoidectomy: a randomized controlled trial. Int J Colorectal Dis. 2002 Jan;17(1):50-3. doi: 10.1007/s003840100342. PMID: 12018455.
112. Peker K, Inal A, Güllü H, Gül D, Sahin M, Ozcan AD, Kılıç K. Comparison of vessel sealing systems with conventional. Iran Red Crescent Med J. 2013 Jun;15(6):488-96. doi: 10.5812/ircmj.10180. Epub 2013 Jun 5. PMID: 24349747; PMCID: PMC3840836.
113. Peng BC, Jayne DG, Ho YH. Randomized trial of rubber band ligation vs. stapled hemorrhoidectomy for prolapsed piles. Dis Colon Rectum. 2003 Mar;46(3):291-7; discussion 296-7. doi: 10.1007/s10350-004-6543-z. PMID: 12626901.
114. Perivoliotis K, Spyridakis M, Zintzaras E, Arnaoutoglou E, Pramateftakis MG, Tepetes K. Non-Doppler hemorrhoidal artery ligation and hemorrhoidopexy combined with pudendal nerve block for the treatment of hemorrhoidal disease: a non-inferiority randomized controlled trial. Int J Colorectal Dis. 2021 Feb;36(2):353-363. doi: 10.1007/s00384-020-03768-8. Epub 2020 Oct 6. PMID: 33025104.
115. Pokharel N, Chhetri RK, Malla B, Joshi HN, Shrestha RK. Haemorrhoidectomy: Ferguson's (closed) vs Milligan Morgan's technique (open). Nepal Med Coll J. 2009 Jun;11(2):136-7. PMID: 19968158.
116. Poskus T, Danys D, Makunaite G, Mainelis A, Mikalauskas S, Poskus E, Jotautas V, Dulskas A, Jasiunas E, Strupas K. Results of the double-blind randomized controlled trial comparing laser hemorrhoidoplasty with sutured mucopexy and excisional hemorrhoidectomy. Int J Colorectal Dis. 2020 Mar;35(3):481-490. doi: 10.1007/s00384-019-03460-6. Epub 2020 Jan 8. PMID: 31912268.
117. Pérez-Vicente F, Arroyo A, Serrano P, Candela F, Sánchez A, Calpena R. Prospective randomised clinical trial of single versus double purse-string stapled mucosectomy in the treatment of prolapsed haemorrhoids. Int J Colorectal Dis. 2006 Jan;21(1):38-43. doi: 10.1007/s00384-004-0720-3. Epub 2005 Apr 21. PMID: 15843940.
118. Qarabaki MA, Mukhashavria GA, Mukhashavria GG, Giorgadze NG. Circular vs. three-quadrant hemorrhoidectomy for end-stage hemorrhoids: short- and long-term outcomes of a prospective randomized trial. J Gastrointest Surg. 2014 Apr;18(4):808-15. doi: 10.1007/s11605-013-2424-x. Epub 2013 Dec 3. PMID: 24297654.
119. Quah HM, Seow-Choen F. Prospective, randomized trial comparing diathermy excision and diathermy coagulation for symptomatic, prolapsed hemorrhoids. Dis Colon Rectum. 2004 Mar;47(3):367-70. doi: 10.1007/s10350-003-0053-2. PMID: 14991499.
120. Qureshi ARZ, Azeem MA, Karim F, Farooq Dar U, Anwar MW, Imtiaz U. Open Hemorrhoidectomy with and without Chemical Sphincterotomy: A Randomized Control Trial. P J M H S. 2015 Apr-Jun;9(2):772-775.
121. Racalbuto A, Aliotta I, Corsaro G, Lanteri R, Di Cataldo A, Licata A. Hemorrhoidal stapler prolapsectomy vs. Milligan-Morgan hemorrhoidectomy: a long-term randomized trial. Int J Colorectal Dis. 2004 May;19(3):239-44. doi: 10.1007/s00384-003-0547-3. Epub 2003 Nov 7. PMID: 14605834.
122. Ravi R, Jayakumar R, Murugadasan P, Kalaranjani V, Nirmala A. A comparative study of open hemorrhoidectomy and rubber band ligation in the treatment of haemorrhoids. Int J Acad Med Pharm. 2023;5(4): 798-801. DOI: 10.47009/jamp.2023.5.4.159
123. Ripetti V, La Vaccara V, Greco S, Arullani A. A Randomized Trial Comparing Stapled Rectal Mucosectomy Versus Open and Semiclosed Hemorrhoidectomy. Dis Colon Rectum. 2015 Nov;58(11):1083-90. doi: 10.1097/DCR.0000000000000454. PMID: 26445182.
124. Rørvik HD, Campos AH, Styr K, Ilum L, McKinstry GK, Brandstrup B, Olaison G. Minimal Open Hemorrhoidectomy Versus Transanal Hemorrhoidal Dearterialization: The Effect on Symptoms: An Open-Label Randomized Controlled Trial. Dis Colon Rectum. 2020 May;63(5):655-667. doi: 10.1097/DCR.0000000000001588. PMID: 31996581.
125. Rowsell M, Bello M, Hemingway DM. Circumferential mucosectomy (stapled haemorrhoidectomy) versus conventional haemorrhoidectomy: randomised controlled trial. Lancet. 2000 Mar 4;355(9206):779-81. doi: 10.1016/s0140-6736(99)06122-x. PMID: 10711924.
126. Saeed MT, Ali Z, Khan SA. Milligan – Morgan (Open) Haemorrhoidectomy VS Rubber band ligation. P J M H S. 2017 Jan-Mar:11(1):396-400.
127. Sakr MF. LigaSure versus Milligan-Morgan hemorrhoidectomy: a prospective randomized clinical trial. Tech Coloproctol. 2010 Mar;14(1):13-7. doi: 10.1007/s10151-009-0549-4. Epub 2009 Dec 9. PMID: 19997953.
128. Schuurman JP, Borel Rinkes IH, Go PM. Hemorrhoidal artery ligation procedure with or without Doppler transducer in grade II and III hemorrhoidal disease: a blinded randomized clinical trial. Ann Surg. 2012 May;255(5):840-5. doi: 10.1097/SLA.0b013e31824e2bb5. PMID: 22504188.
129. Senagore AJ, Singer M, Abcarian H, Fleshman J, Corman M, Wexner S, Nivatvongs S; Procedure for Prolapse and Hemmorrhoids (PPH) Multicenter Study Group. A prospective, randomized, controlled multicenter trial comparing stapled hemorrhoidopexy and Ferguson hemorrhoidectomy: perioperative and one-year results. Dis Colon Rectum. 2004 Nov;47(11):1824-36. doi: 10.1007/s10350-004-0694-9. Erratum in: Dis Colon Rectum. 2005 Feb;48(2):400. Erratum in: Dis Colon Rectum. 2005 May;48(5):1099. PMID: 15622574.
130. Shabahang H, Maddah G, Mofidi A, Nooghabi MJ, Khaniki SH. A Randomized Clinical Trial of Laser Hemorrhoidoplasty vs Milligan and Morgan Hemorrhoidectomy. World J Lap Surg 2019; 12 (2):59-63. DOI: 10.5005/jp-journals-10033-1373
131. Shahmoradi MK, Mehri J, Taheri HR. Comparison of hemorrhoidectomy using harmonic scalpel and electrocautery: A randomized controlled trial. International Journal of Surgery Open. 2020;27:39-42. ISSN 2405-8572. https://doi.org/10.1016/j.ijso.2020.10.006.
132. Shaikh AR, Dalwani AG, Soomro N. An evaluation of Milligan-Morgan and Ferguson procedures for haemorrhoidectomy at Liaquat University Hospital Jamshoro, Hyderabad, Pakistan. Pak J Med Sci. 2013 Jan;29(1):122-7. doi: 10.12669/pjms.291.2858. PMID: 24353522; PMCID: PMC3809167.
133. Shalaby R, Desoky A. Randomized clinical trial of stapled versus Milligan-Morgan haemorrhoidectomy. Br J Surg. 2001 Aug;88(8):1049-53. doi: 10.1046/j.0007-1323.2001.01830.x. PMID: 11488788.
134. Shanmugam V, Muthukumarasamy G, Cook JA, Vale L, Watson AJ, Loudon MA. Randomized controlled trial comparing rubber band ligation with stapled haemorrhoidopexy for Grade II circumferential haemorrhoids: long-term results. Colorectal Dis. 2010 Jun;12(6):579-86. doi: 10.1111/j.1463-1318.2009.01841.x. PMID: 19508542.
135. Shehata AM, Saleh AF, El-Heeny AAEH. Clinical Outcome after Doppler-Guided Hemorrhoidal Artery Ligation and Rubber Band Ligation for Treatment of Primary Symptomatic Hemorrhoids. Indian J Surg 81, 332–337 (2019). https://doi.org/10.1007/s12262-018-1797-1
136. Shoukat H, Iqbal M, Ullah S, Mirza A, Farooq Dar U, Farooq Dar U. Comparison of hemorrhoidectomy using bipolar diathermy vs harmonic scalpel. P J M H S. 2016 Apr-Jun:10(2):489-491
137. Shukla S, Maheshwari A, Tiwari B. Randomized Trial of Open Hemorrhoidectomy Versus Stapled Hemorrhoidectomy for Grade II/III Hemorrhoids. Indian J Surg 80, 574–579 (2018). https://doi.org/10.1007/s12262-017-1670-7
138. Siddiqui K, Choudhary H, Aslam U. Short and Long-Term Results of Stapled Vs Conventional Hemorrhoidectomy ; Our Experience. 2016. P J M H S. 2015 Apr-Jun;9(2):701-706.
139. Smyth EF, Baker RP, Wilken BJ, Hartley JE, White TJ, Monson JR. Stapled versus excision haemorrhoidectomy: long-term follow up of a randomised controlled trial. Lancet. 2003 Apr 26;361(9367):1437-8. doi: 10.1016/S0140-6736(03)13106-6. PMID: 12727401.
140. Song X, Sun W, Bao Y, Tu J, Zhang T. Outcome of a modified Park's submucosal hemorrhoidectomy versus Milligan-Morgan for grade III-IV circumferential prolapsed hemorrhoids. Asian J Surg. 2022 Nov;45(11):2208-2213. doi: 10.1016/j.asjsur.2021.11.032. Epub 2021 Nov 25. PMID: 34840045.
141. Srinivas L, Hemanth G. Clinical Evaluation of Outcome of Open and Closed Haemorrhoidectomy. Int J of Pharmaceutical and Clinical Research. 2023;15(6):1785-1792.
142. Talha A, Bessa S, Abdel Wahab M. Ligasure, Harmonic Scalpel versus conventional diathermy in excisional haemorrhoidectomy: a randomized controlled trial. ANZ J Surg. 2017 Apr;87(4):252-256. doi: 10.1111/ans.12838. Epub 2014 Sep 11. PMID: 25214362.
143. Tan JJ, Seow-Choen F. Prospective, randomized trial comparing diathermy and Harmonic Scalpel hemorrhoidectomy. Dis Colon Rectum. 2001 May;44(5):677-9. doi: 10.1007/BF02234565. PMID: 11357028.
144. Teksoz S, Aytac E, Yavuz N, Tortum OB, Ozcan M, Erguney S, Bukey Y. Comparison of a Vessel Sealing System with a Conventional Technique in Hemorrhoidectomy. Balkan Med J 2011; 28: 189-192. DOI: 10.5174/tutfd.2010.03074.2
145. Thaha MA, Campbell KL, Kazmi SA, Irvine LA, Khalil A, Binnie NR, Hendry WS, Walker A, Staines HJ, Steele RJ. Prospective randomised multi-centre trial comparing the clinical efficacy, safety and patient acceptability of circular stapled anopexy with closed diathermy haemorrhoidectomy. Gut. 2009 May;58(5):668-78. doi: 10.1136/gut.2008.151266. Epub 2008 Dec 17. PMID: 19091821.
146. Thorbeck CV, Montes MF. Haemorrhoidectomy: randomised controlled clinical trial of Ligasure compared with Milligan-Morgan operation. Eur J Surg. 2002;168(8-9):482-4. doi: 10.1080/110241502321116497. PMID: 12549689.
147. Trenti L, Biondo S, Espin-Basany E, Barrios O, Sanchez-Garcia JL, Landaluce-Olavarria A, Bermejo-Marcos E, Garcia-Martinez MT, Alias Jimenez D, Jimenez F, Alonso A, Manso MB, Kreisler E; THDLIGA-RCT Study Group. Transanal Hemorrhoidal Dearterialization With Mucopexy Versus Vessel-Sealing Device Hemorrhoidectomy for Grade III to IV Hemorrhoids: Long-term Outcomes From the THDLIGA Randomized Controlled Trial. Dis Colon Rectum. 2023 Aug 1;66(8):e818-e825. doi: 10.1097/DCR.0000000000002272. Epub 2022 Mar 1. PMID: 35239526.
148. Tsunoda A, Sada H, Sugimoto T, Kano N, Kawana M, Sasaki T, Hashimoto H. Randomized controlled trial of bipolar diathermy vs ultrasonic scalpel for closed hemorrhoidectomy. World J Gastrointest Surg. 2011 Oct 27;3(10):147-52. doi: 10.4240/wjgs.v3.i10.147. PMID: 22110846; PMCID: PMC3220727.
149. Tsunoda A, Takahashi T, Kusanagi H. A prospective randomized trial of transanal hemorrhoidal dearterialization with mucopexy versus ultrasonic scalpel hemorrhoidectomy for grade III hemorrhoids. Tech Coloproctol. 2017 Aug;21(8):657-665. doi: 10.1007/s10151-017-1673-1. Epub 2017 Sep 4. PMID: 28871428.
150. Van de Stadt J, D'Hoore A, Duinslaeger M, Chasse E, Penninckx F; Belgian Section of Colorectal Surgery Royal Belgian Society for Surgery. Long-term results after excision haemorrhoidectomy versus stapled haemorrhoidopexy for prolapsing haemorrhoids; a Belgian prospective randomized trial. Acta Chir Belg. 2005 Feb;105(1):44-52. PMID: 15790202.
151. Venara A, Podevin J, Godeberge P, Redon Y, Barussaud ML, Sielezneff I, Queralto M, Bourbao C, Chiffoleau A, Lehur PA; LigaLongo Study Group. A comparison of surgical devices for grade II and III hemorrhoidal disease. Results from the LigaLongo Trial comparing transanal Doppler-guided hemorrhoidal artery ligation with mucopexy and circular stapled hemorrhoidopexy. Int J Colorectal Dis. 2018 Oct;33(10):1479-1483. doi: 10.1007/s00384-018-3093-8. Epub 2018 May 28. PMID: 29808305.
152. Vijayaraghavalu S, Prasad R G, Rajkumar S. The Role of Lateral Internal Sphincterotomy in Haemorrhoidectomy: A Study in a Tertiary Care Center. Cureus. 2021 Jun 13;13(6):e15630. doi: 10.7759/cureus.15630. PMID: 34306842; PMCID: PMC8277982.
153. Wadhawan G, Soni BM, Vyas KC. Ligasure haemorrhoidectomy in comparison with conventional haemorrhoidectomy- a case study on 100 patients. J. Evolution Med. Dent. Sci. 2018;7(37):4091-4094, DOI: 10.14260/jemds/2018/915
154. Wang JY, Lu CY, Tsai HL, Chen FM, Huang CJ, Huang YS, Huang TJ, Hsieh JS. Randomized controlled trial of LigaSure with submucosal dissection versus Ferguson hemorrhoidectomy for prolapsed hemorrhoids. World J Surg. 2006 Mar;30(3):462-6. doi: 10.1007/s00268-005-0297-1. PMID: 16479346.
155. Wang JY, Tsai HL, Chen FM, Chu KS, Chan HM, Huang CJ, Hsieh JS. Prospective, randomized, controlled trial of Starion vs Ligasure hemorrhoidectomy for prolapsed hemorrhoids. Dis Colon Rectum. 2007 Aug;50(8):1146-51. doi: 10.1007/s10350-007-0260-3. PMID: 17587087.
156. Wang ZG, Zhang Y, Zeng XD, Zhang TH, Zhu QD, Liu DL, Qiao YY, Mu N, Yin ZT. Clinical observations on the treatment of prolapsing hemorrhoids with tissue selecting therapy. World J Gastroenterol. 2015 Feb 28;21(8):2490-6. doi: 10.3748/wjg.v21.i8.2490. PMID: 25741159; PMCID: PMC4342928.
157. Watson AJ, Hudson J, Wood J, Kilonzo M, Brown SR, McDonald A, Norrie J, Bruhn H, Cook JA; eTHoS study group. Comparison of stapled haemorrhoidopexy with traditional excisional surgery for haemorrhoidal disease (eTHoS): a pragmatic, multicentre, randomised controlled trial. Lancet. 2016 Nov 12;388(10058):2375-2385. doi: 10.1016/S0140-6736(16)31803-7. Epub 2016 Oct 7. Erratum in: Lancet. 2016 Nov 12;388(10058):2354. doi: 10.1016/S0140-6736(16)32059-1. PMID: 27726951; PMCID: PMC5269572.
158. Yang R, Migikovsky B, Peicher J, Laine L. Randomized, prospective trial of direct current versus bipolar electrocoagulation for bleeding internal hemorrhoids. Gastrointest Endosc. 1993 Nov-Dec;39(6):766-9. doi: 10.1016/s0016-5107(93)70261-8. PMID: 8293898.
159. Yang H, Shi Z, Chen W, Chen T, Ding P, Wang J, Gao J. Modified ligation procedure for prolapsed haemorrhoids versus stapled haemorrhoidectomy for the management of symptomatic haemorrhoids (MoLish): randomized clinical trial. BJS Open. 2022 May 2;6(3):zrac064. doi: 10.1093/bjsopen/zrac064. PMID: 35552375; PMCID: PMC9099087.
160. You SY, Kim SH, Chung CS, Lee DK. Open vs. closed hemorrhoidectomy. Dis Colon Rectum. 2005 Jan;48(1):108-13. doi: 10.1007/s10350-004-0794-6. PMID: 15690666.
161. Zaher T, Ibrahim I, Ibrahim A. Endoscopic band ligation of internal haemorrhoids versus stapled haemorrhoidopexy in patients with portal hypertension. Arab J Gastroenterol. 2011 Mar;12(1):11-4. doi: 10.1016/j.ajg.2011.01.009. PMID: 21429448.
162. Zhai M, Zhang YA, Wang ZY, Sun JH, Wen J, Zhang Q, Li JD, Wu YZ, Zhou F, Xu HL. A Randomized Controlled Trial Comparing Suture-Fixation Mucopexy and Doppler-Guided Hemorrhoidal Artery Ligation in Patients with Grade III Hemorrhoids. Gastroenterol Res Pract. 2016;2016:8143703. doi: 10.1155/2016/8143703. Epub 2016 Mar 15. PMID: 27066071; PMCID: PMC4811093.
